# Snowmaking Infrastructure: A Selective Barrier or Potential Vector for Antibiotic Residues and Resistance Determinants in Mountain Catchments

**DOI:** 10.3390/ijms27188172

**Published:** 2026-09-14

**Authors:** Natalia Czernecka-Borchowiec, Klaudia Stankiewicz, Klaudia Bulanda, Piotr Boroń, Justyna Prajsnar, Anna Kopacz, Zofia Schejbal, Anna Lenart-Boroń

**Affiliations:** 1 Section of Microbiology, Scientific Circle of Biotechnologists, University of Agriculture in Kraków, 31-120 Kraków, Poland; natalia.czernecka2@student.urk.edu.pl (N.C.-B.); anna.kopacz@student.urk.edu.pl (A.K.); zofia.schejbal@student.urk.edu.pl (Z.S.); 2 Department of Pharmaceutical Microbiology, Faculty of Pharmacy, Jagiellonian University Medical College, Medyczna 9 Street, 30-688 Kraków, Poland; klaudia.stankiewicz@uj.edu.pl; 3 Department of Forest Ecosystems Protection, Faculty of Forestry, University of Agriculture in Kraków, 29 Listopada Ave. 46, 31-425 Kraków, Poland; klaudia.bulanda@student.urk.edu.pl (K.B.); piotr.boron@urk.edu.pl (P.B.); 4 Jerzy Haber Institute of Catalysis and Surface Chemistry, Polish Academy of Sciences, Niezapominajek 8, 30-239 Kraków, Poland; justyna.prajsnar@ikifp.edu.pl; 5 Department of Microbiology and Biomonitoring, University of Agriculture in Kraków, Mickiewicza Ave. 24/28, 30-059 Kraków, Poland

**Keywords:** alpine catchments, antibiotics, antibiotic resistance genes (ARGs), antibiotic resistant bacteria (ARB), next-generation sequencing, one health, technical snowmaking

## Abstract

Snowmaking systems are increasingly used to serve winter tourism in mountain regions, yet their role in the environmental dissemination of antimicrobial resistance (AMR) remains poorly understood. Here, we investigated how snowmaking infrastructure affects the transport, retention and reduction of antibiotic residues, antibiotic resistance genes (ARGs) and phenotypically resistant bacteria along two interconnected water chains feeding technical snow production in mountain catchments. Over two winter seasons (2023/24 and 2024/25), we quantified a panel of clinically relevant antibiotics, ARGs and resistance phenotypes in wastewater effluent, intake water, reservoirs, technical snow, aged snow, meltwater and receiving streams; culturable bacteria, resistance phenotypes and ARGs were additionally assessed in reservoir sediments and snow cannon filter material. Wastewater and intake water exhibited the highest antibiotic loads and ARG richness, dominated by fluoroquinolones, macrolides and β-lactam resistance determinants. Reservoirs accumulated substantial amounts of antibiotics but their reduction occurred further along the snowmaking chain. Fresh technical snow still contained multiple antibiotics and ARGs, and harbored resistant *Enterobacteriaceae*, indicating that snowmaking systems can redistribute AMR determinants into the snowpack. Aged snow, meltwater and receiving streams showed nearly complete loss of antibiotic residues and reduced ARG richness, due to processes of snow transformation and downstream transport. By integrating chemical, molecular and phenotypic data with the technical layout of the infrastructure, we identify specific components as AMR retention hotspots and clarify where barrier functions are most effective. Our findings demonstrate that snowmaking infrastructure can both concentrate and distribute antibiotic residues and resistance determinants in mountain catchments, highlighting the need to consider snowmaking systems in environmental AMR risk assessments and One Health frameworks.

## 1. Introduction

Antimicrobial resistance (AMR) is one of the most urgent threats to global public health, projected to cause 10 million deaths annually by 2050 if it is not dealt with [1]. Although antibiotic resistance originated as a natural phenomenon, it has become the most significant threat in clinical settings. From these clinical settings, resistant bacteria escaped to become an equally important environmental issue. It has been confirmed that aquatic environments are among the most significant reservoirs and transmission routes for antibiotic residues, antibiotic resistance genes (ARGs) and resistant bacteria (ARB) [2,3,4]. Wastewater treatment plants (WWTPs) are the central element of this process. After receiving mixtures of pharmaceutical agents, microorganisms (including antibiotic resistant ones) and genetic determinants of antibiotic resistance from human and veterinary sources, WWTPs release these incompletely removed contaminants into receiving water bodies [5,6]. From these source points, antibiotics and AMR determinants enter the aquatic environment, where they interact with environmental microbial communities, undergo dilution, sorption, transformation and selective enrichment, and ultimately reach ecosystems and human populations through multiple exposure pathways [7,8].

Mountain and alpine water systems are unique elements of the water network, but they have received very little attention compared with the importance of their role. Mountain catchments are generally subjected to low anthropogenic pressure, characterized by low temperatures, high UV irradiance and short hydraulic residence times and, for this reason, they have long been considered pristine, self-purifying environments, with negligible AMR loads [9,10]. However, this assumption has been questioned by a number of studies conducted in the Alps and the Carpathians, in which antibiotic residues, ARGs and ARB have been detected in high altitude streams, lakes or even mountain springs [9,11,12]. Nevertheless, mountain environments remain substantially underrepresented in the global AMR literature relative to lowland agricultural and urban systems [6,13,14], and thus the specific pathways through which resistance determinants enter, move through and exit these catchments remain poorly characterized.

Within mountain catchments, artificial snowmaking represents a rapidly expanding but entirely overlooked potential pathway for AMR dissemination. Snowmaking systems are now implemented across the majority of ski resorts not only in Europe or North America but also in a variety of countries where global winter competitions take place. As of 2024, more than 75% of Alpine ski resorts (75% in Italy and 90% in Austria) rely on technical snow to compensate for climate change-driven decline in natural snowfall [15]. The process relies on large volumes of water being pumped from water sources, pressurized, atomized and sprayed onto ski slopes to form the snowpack, while the critical element of the system is the source water for snowmaking. It is frequently drawn from watercourses affected by or directly receiving WWTP effluents [16,17], meaning that snowmaking chains (from intakes through storage reservoirs, water filtration, atomization and snow deposition) may function as previously unrecognized routes through which wastewater-derived antibiotic residues, ARGs and ARB are concentrated, transformed and ultimately released into mountain catchments via meltwater. Despite the scale of snowmaking operations across mountain regions, no study has systematically investigated the fate of AMR determinants across the complete snowmaking infrastructure.

There are elements of snowmaking infrastructure whose roles can be ambiguous in the context of AMR. Storage reservoirs may act as concentration points through evaporation, sedimentation and differential sorption, but their more definitive elements, such as aeration, filtration or UV light-mediated disinfection, can contribute to elimination or decrease of antimicrobial agents, ARB and ARGs. Mechanical filtration components on one hand prevent the spread of contaminants, but on the other may accumulate biofilms that harbor ARB and ARGs. The snowmaking process in the snow cannons, involving freeze-atomization, subjects the contaminants to rapid temperature change, shear stress and aeration, the selective effects of which are unknown. Once deposited, the technical snow undergoes transformation over several weeks/months, during which UV light exposure, freeze–thaw cycling and microbial succession may further attenuate or transform contaminants. Finally, snowmelt constitutes a seasonal rapid input to receiving streams, the AMR burden of which has never been quantified in a snowmaking context. Whether snowmaking infrastructure ultimately acts as an AMR barrier or its vector is an open question with direct implications for alpine catchment management.

In this study, we address this gap through a two-season investigation of AMR fate across a snowmaking system of a ski resort located in the Carpathian region. Based on our previous studies, we extend the analysis to identify AMR retention hotspots and attenuation stages within the infrastructure. Our study comprises (i) quantification of 21 clinically and veterinary relevant antibiotic compounds across the complete snowmaking chain using UHPLC-MS/MS; (ii) assessment of ARG richness by targeted PCR-based screening of 37 resistance genes; (iii) phenotypic resistance profiling of 176 bacterial isolates across nine system compartments; (iv) 16S rRNA amplicon sequencing to characterize microbial community diversity independently of cultivation; and (v) multivariate integration of all data.

By tracking AMR determinants simultaneously at the chemical, molecular, phenotypic and community levels across two snowmaking seasons, this study provides the first integrated evidence base for understanding how snowmaking infrastructure modulates AMR fate in mountain catchments, with implications for environmental risk assessment and One Health management of mountain water systems.

## 2. Results

### 2.1. Bacterial Indicators of Contamination

**Figure 1:**
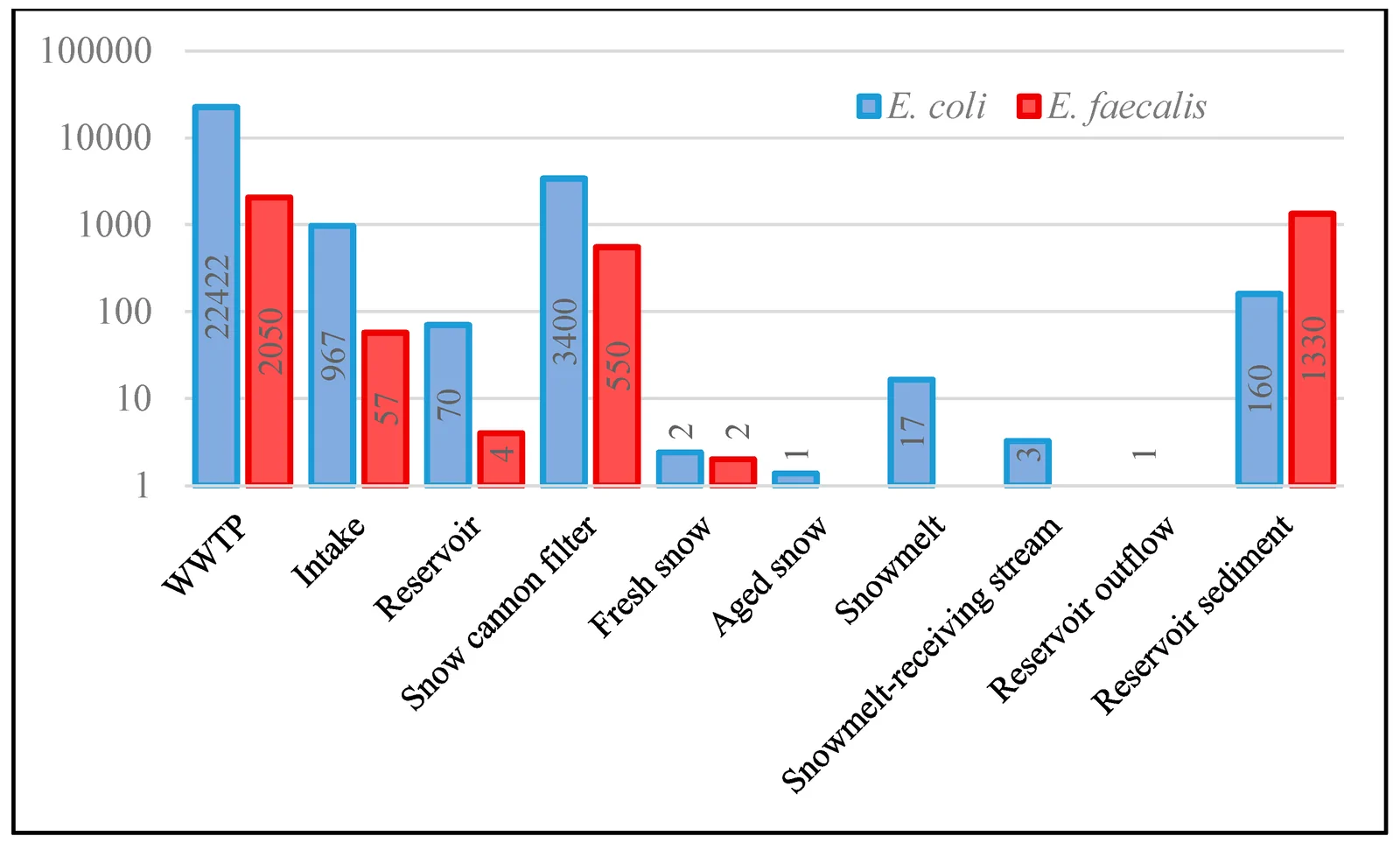
log-10-transformed counts of *E. coli* (blue) and *E. faecalis* (red) along the snowmaking chains (CFU/100 mL of water and CFU/ 1g of sediment, values averaged for all assessments).

*Escherichia coli* and *Enterococcus faecalis* counts along both snowmaking chains showed a gradient of lower counts from wastewater effluent to receiving waters (Figure 1, Supplementary Table S1), consistent with progressive dilution and retention along the infrastructure, though the compartments were sampled on different dates and do not represent the same water parcel. WWTP effluent contained means of 22,422 CFU/100 mL for *E. coli* and 2050 CFU/100 mL for *E. faecalis*, while water intake samples showed clearly lower counts (967 and 57, respectively), which were then also found to be lower in the reservoir samples. Snow cannon filters retained bacteria (elevated *E. coli* and *E. faecalis* numbers relative to reservoir water), with fresh and aged technical snow again showing lower counts. The number of *E. coli* was reduced from 967 CFU/100 mL in water intake for technical snowmaking to 1 CFU/100 mL in aged snow and the number of *E. faecalis* dropped from 57 CFU/100 mL in intake water to none in technical snow. As indicated on the graph, the number of culturable *E. coli* increases in snowmelt. In addition, relatively higher numbers of *E. coli* have been detected in technical snowing-related residues (i.e., snow cannon filter material and reservoir sediments). As residue samples are not unexpected, because they result from the concentration of the material suspended in water, the increased amount of *E. coli* in snowmelt must result from external sources, not associated with snowmaking.

Bacterial counts differed significantly among compartments for *E. coli* (Kruskal–Wallis H(8) = 22.90, *p* = 0.0035), *Salmonella* (*p* < 0.001) and *Staphylococcus* (*p* = 0.0017), but not for *Enterococcus* (*p* = 0.036), detailed data are presented in Supplementary Table S1.

### 2.2. Microbial Community Diversity Along the Snowmaking Chains

**Figure 2:**
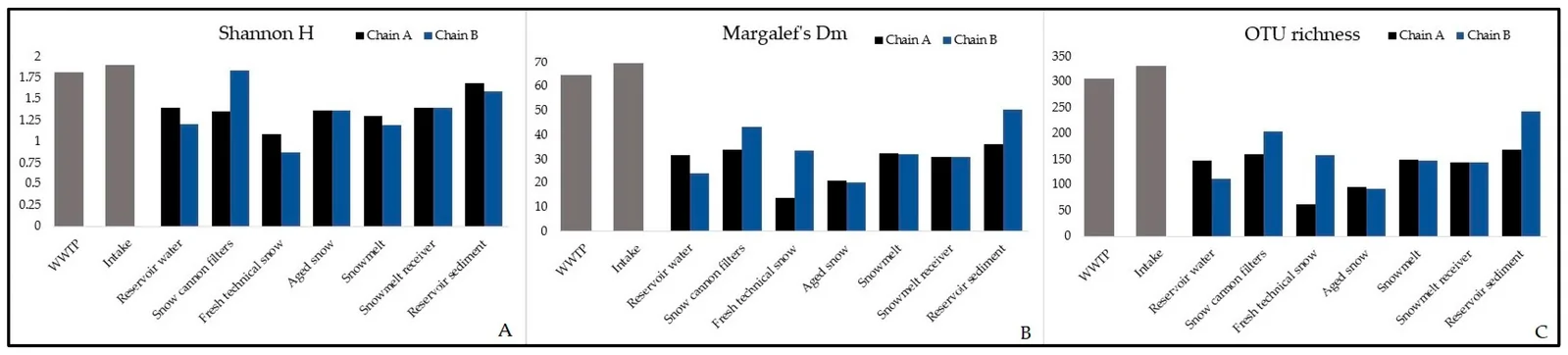
Alpha diversity indices ((**A**): Shannon H, (**B**): Margalef’s Dm, (**C**): observed OTU richness) of bacterial communities across snowmaking-chain compartments, based on 16S rRNA amplicon data. Values represent a single sequencing run per compartment/chain. WWTP effluent and intake water are common to both chains.

Observed OTU richness and Shannon diversity were highest at the WWTP effluent (SR = 296, H = 1.753) and water intake (SR = 321, H = 1.833) (Supplementary Table S1, Figure 2), consistent with these compartments receiving highly complex, mixed microbial inputs from municipal wastewater. Dominance was correspondingly low at both sites (λ < 0.04), indicating structurally even communities. Reservoir water showed reduced observed OTU richness when compared with the intake waters (reservoir A: SR = 148, H = 1.402; reservoir B: SR = 111, H = 1.211), accompanied by a moderate increase in dominance (λ = 0.073–0.149).

Snow cannon filters differed clearly between chains. Chain A filters (A1: H = 1.415, SR = 179; A2: H = 1.288, SR = 141) showed reduced diversity in comparison to the intake water, with elevated dominance (λ = 0.113–0.117), consistent with selective bacterial retention on filter surfaces. In contrast, the microbial diversity of filter B was similar to the intake (H = 1.831, SR = 205, λ = 0.026) and had the highest evenness values among any infrastructure component. This divergence in community structure is consistent with the ARG data: the more diverse and even filter B community corresponded to lower ARG retention, while the more dominated filter A was associated with elevated ARG richness (Section 3.5). Fresh technical snow showed clear chain-specific patterns. In chain A, fresh snow exhibited the lowest observed OTU richness of all sampled compartments (SR = 63, H = 1.086, λ = 0.196). The fresh snow of chain B presented an apparent anomaly: observed OTU richness was comparatively high (SR = 159) yet Shannon index was the lowest recorded in the entire study (H = 0.878) and dominance was extreme (λ = 0.402). Snow ageing further increased the community evenness relative to fresh snow. Shannon diversity rose from H = 1.086 in fresh snow A to H = 1.367 in aged snow A, and from H = 0.878 in fresh snow B to H = 1.368 in aged snow B, despite both aged snow sites retaining low observed OTU richness (SR = 96 and 93, respectively).

Snowmelt and receiving stream samples showed intermediate diversity (H = 1.200–1.403, SR = 145–151). Finally, reservoir sediments demonstrated nearly highest observed OTU richness among the compartments (reservoir A sediment: SR = 169, H = 1.688; reservoir B sediment: SR = 243, H = 1.592), with low dominance values (λ = 0.041–0.066) comparable to the WWTP and intake sites.

### 2.3. Antibiotic Residues Along the Snowmaking Chain

**Figure 3:**
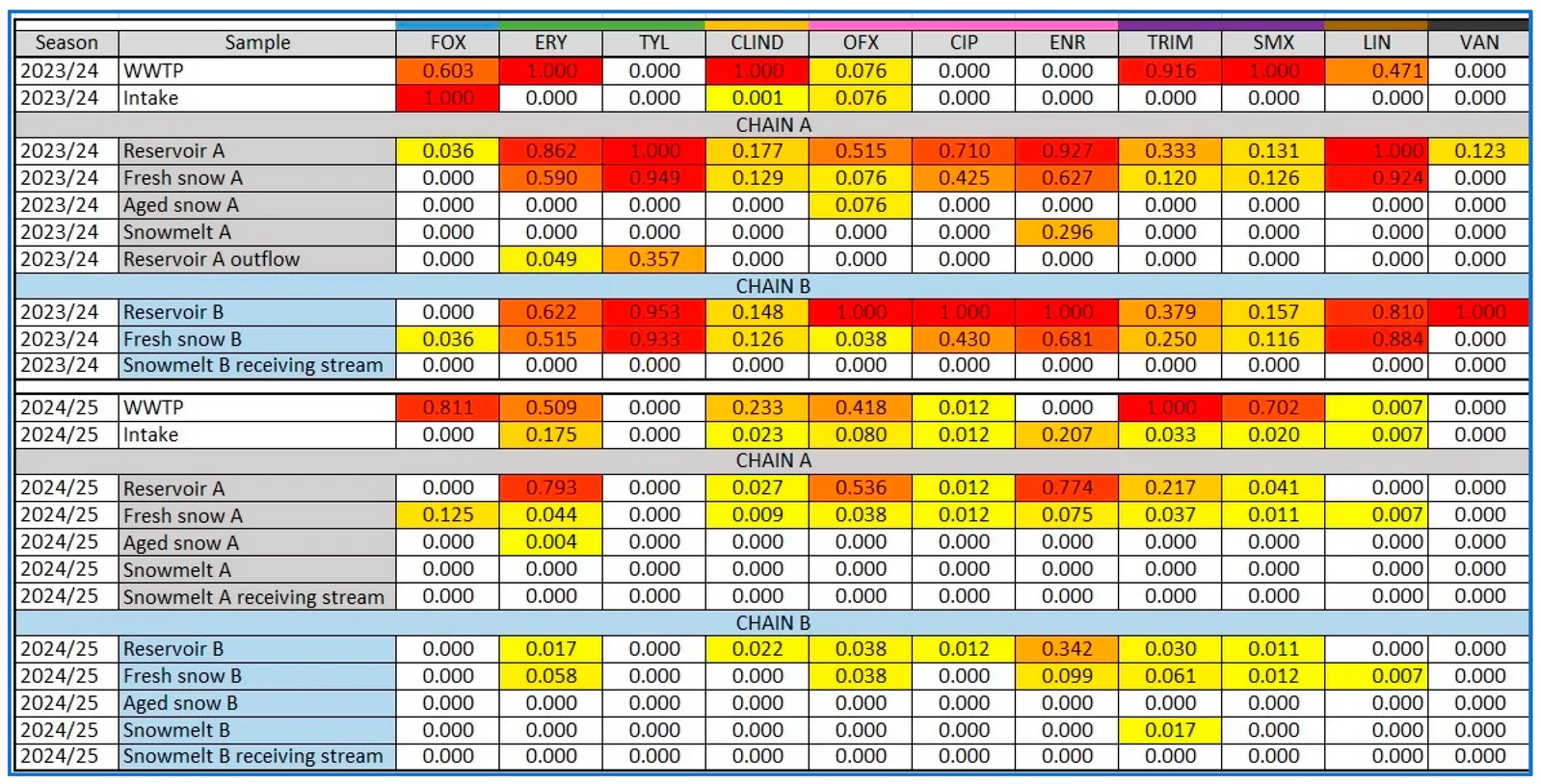
Heatmap of normalized peak antibiotic concentrations across the snowmaking system in two winter seasons (2023/24 and 2024/25). Values represent relative intensities (0–1) normalized to the maximum concentration detected for each antibiotic. Cell shading reflects the normalized concentration value (0–1) for each compound and compartment, ranging from white (0.000, not detected) through yellow/orange (intermediate values) to red (values approaching 1.000, the maximum normalized concentration for that compound). The system shows strong reduction of antibiotic residues from wastewater and intake water through reservoirs and technical snow, with nearly complete disappearance in aged snow and meltwater. Sediment and snow cannon filter material, which were not subjected to chemical analysis, are not included.

**Figure 4:**
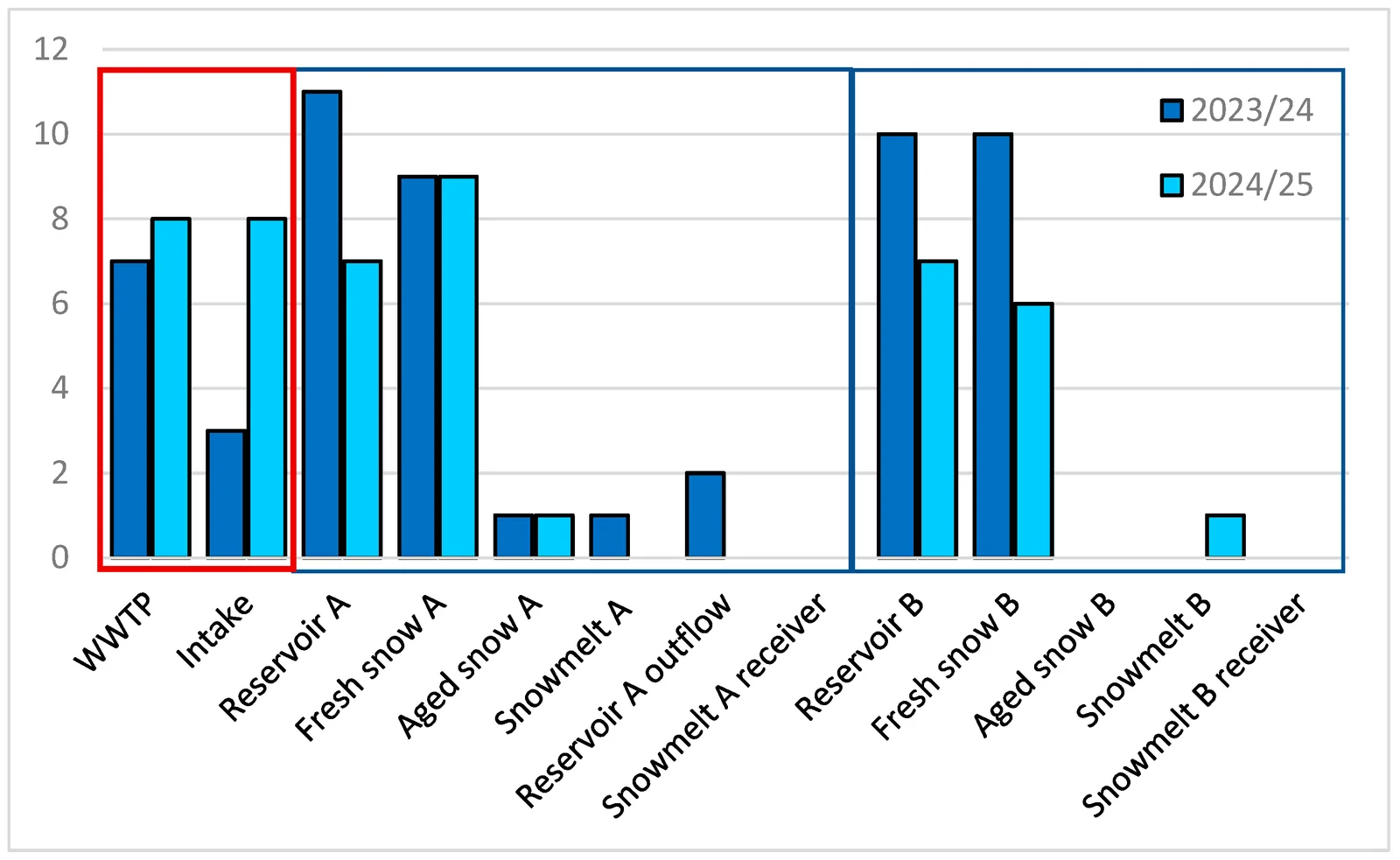
Antibiotic richness across the snowmaking system in two winter seasons (2023/24 and 2024/25). Richness was defined as the number of individual antibiotics detected at each sampling point (0–11). The red box indicates sampling points shared between both snowmaking chains (WWTP effluent and intake water), while blue boxes delineate compartments specific to Chain A and Chain B, respectively. Wastewater and intake water showed the highest richness, followed by reservoirs and fresh technical snow. Only water and snow matrices were chemically analyzed; sediment and filter material are not included.

Eleven antibiotic compounds of seven pharmacological classes, i.e., β-lactams (cefoxitin, FOX), macrolides (erythromycin, ERY; tylosin, TYL), lincosamides (clindamycin, CLIND), fluoroquinolones (ofloxacin, OFX; ciprofloxacin, CIP; enrofloxacin, ENR), a dihydrofolate reductase inhibitor (trimethoprim, TRIM), sulfonamides (sulfamethoxazole SMX), oxazolidinones (linezolid, LIN) and glycopeptides (vancomycin, VAN), were detected in the sampled water and snow matrices in both seasons; filter material and sediments were not analyzed chemically. Normalized peak intensities and compound richness are presented in Figure 3 and Figure 4, respectively.

Trimethoprim and sulfamethoxazole concentrations differed significantly among compartments (H(6) = 22.60 and 21.82, *p* < 0.001), driven by consistently higher WWTP effluent inputs. Importantly, of the eleven antibiotics measured, only trimethoprim and sulfamethoxazole showed a clear, statistically confirmed decline along the snowmaking chain, making them the strongest chemical evidence for wastewater-derived contamination in this study. The remaining compounds were either present at low, statistically indistinguishable levels throughout, or too inconsistently detected to draw firm conclusions. Total antibiotic concentration correlated significantly and positively with ARG richness (Pearson r = 0.61, *p* < 0.001, n = 52), driven primarily by sulfamethoxazole (r = 0.77, *p* < 0.001) and trimethoprim (r = 0.66, *p* < 0.001), i.e., the same two compounds that differed significantly between compartments. No significant correlation was found between antibiotic concentration and phenotypic resistance percentages (all *p* > 0.07, Supplementary Table S1).

Antibiotic richness was the highest at WWTP effluent (up to eight compounds detected in 2024/25) and water intake (Figure 3 and Figure 4). Cefoxitin reached its maximum normalized intensity at water intake (1.0) in the 2023/24 season, consistent with the direct input of β-lactam antibiotics in treated wastewater entering the catchment stream. WWTP effluent showed the highest concentrations of erythromycin (1.0 in 2023/24), clindamycin (1.0 in 2023/24), sulfamethoxazole (1.0 in 2023/24, 1.0 in 2024/25), trimethoprim (0.92 in 2023/24) and linezolid (0.47 in 2023/24), confirming that the wastewater treatment plant is a multi-class antibiotic source for the snowmaking water supply.

Reservoirs, on the other hand, showed an antibiotic accumulation pattern. In the 2023/24 season, in reservoir A there was the maximum normalized concentration of tylosin (1.0), linezolid (1.0) and almost maximum concentrations of fluoroquinolones (OFX 0.51, CIP 0.71, ENR 0.93) and erythromycin (0.86), values that exceeded those recorded in WWTP effluent for these compounds (Figure 3). Reservoir B in the same season showed maximum concentrations of ofloxacin (1.0), ciprofloxacin (1.0), enrofloxacin (1.0) and vancomycin (1.0). In the 2024/25 season, the concentrations of some antimicrobials were lower than in the input water, while those of erythromycin, ofloxacin, and enrofloxacin remained higher than the ones recorded for the WWTP.

Fresh technical snow still contained multiple antibiotic compounds in both chains and both seasons. In chain A (2023/24), tylosin (0.95), linezolid (0.92), enrofloxacin (0.63) and erythromycin (0.59) were detected at moderate/high normalized concentrations, indicating that the atomization and freezing process during snowmaking does not fully eliminate antibiotic residues. Chain B fresh snow showed a comparable antibiotic profile (tylosin 0.93, linezolid 0.88, enrofloxacin 0.68). In the 2024/25 season, intensities in fresh snow were lower for all compounds, consistent with the reduced input concentrations observed in that season’s reservoir water, though enrofloxacin, erythromycin and cefoxitin remained detectable above background.

Aged snow constituted a significant reduction stage for antibiotic residues. In both seasons and both chains, the vast majority of targeted compounds dropped to non-detectable levels (Figure 3 and Figure 4). The exceptions were a trace erythromycin signal in aged snow A (2024/25; normalized intensity 0.004) and ofloxacin in aged snow A (2023/24; 0.076). Snowmelt and receiving stream samples were nearly free of antibiotics in both seasons, with the exception of a low enrofloxacin signal in chain A snowmelt (2023/24; 0.30) and trimethoprim in chain B snowmelt (2024/25; 0.017).

The overall pattern of compound richness (Figure 4) reflected the intensity data: richness was the highest at WWTP and reservoir A (up to 10–11 compounds), lower in fresh snow (6–9 compounds), and the lowest (1–2 compounds) in aged snow, with no compounds detected in snowmelt and receiving stream samples. The two seasons showed consistent richness profiles, though the 2023/24 season exhibited higher richness and intensity at reservoir and fresh snow stages, likely reflecting inter-annual variation in wastewater effluent composition and in the upstream sites.

### 2.4. Phenotypic Resistance of Isolated Bacteria

A total of 176 bacterial isolates were obtained from all system compartments, two seasons and both chains, and these comprised primarily *Enterobacteriaceae* (n = 57), *Aeromonas* spp. (n = 81), *Acinetobacter* spp. (n = 11) and *Pseudomonas* spp., (n = 8) with a few isolates of environmental bacilli (n = 14) and cocci (n = 5) (Table 1, Supplementary Table S1).

**Figure 5:**
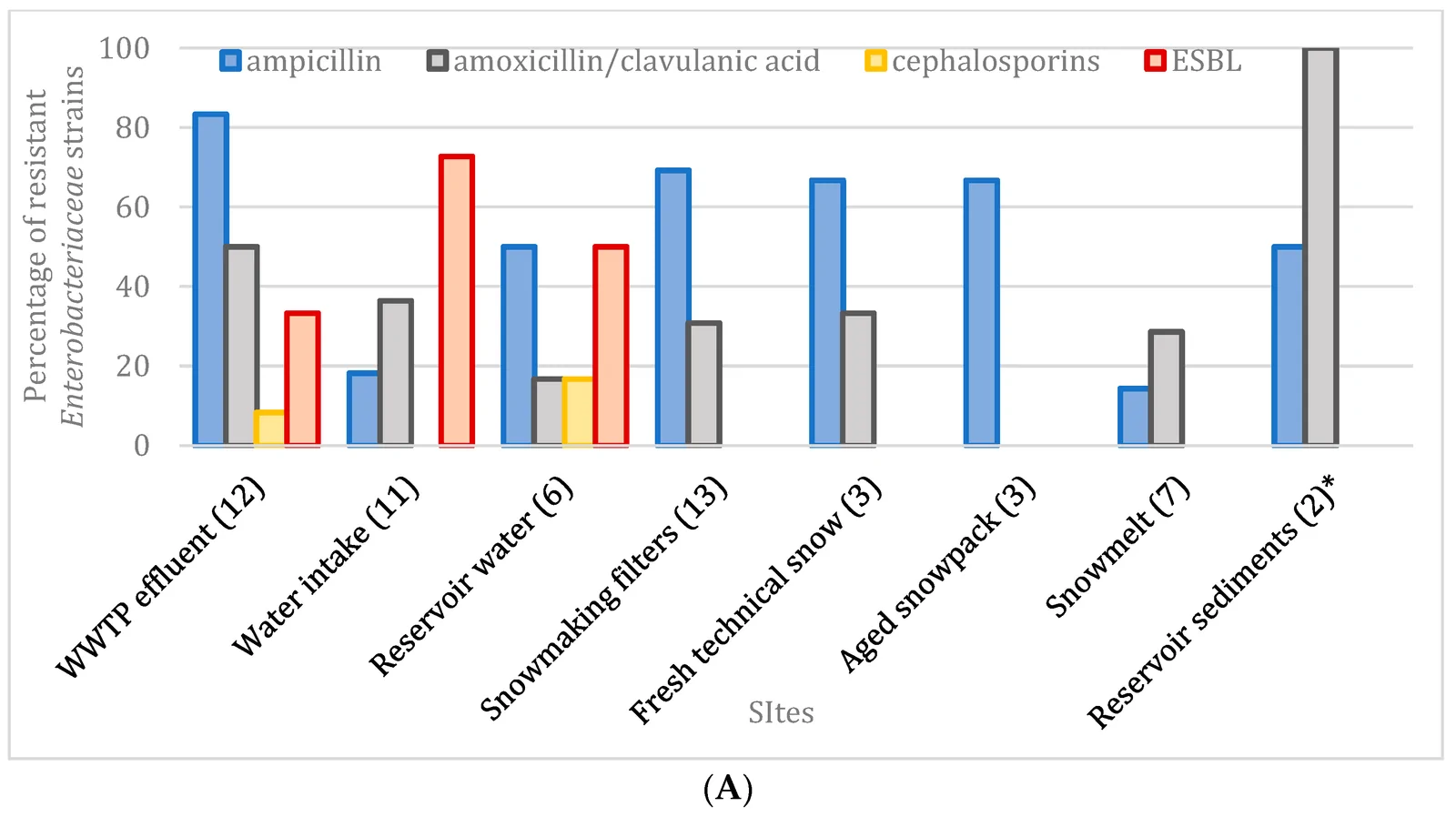

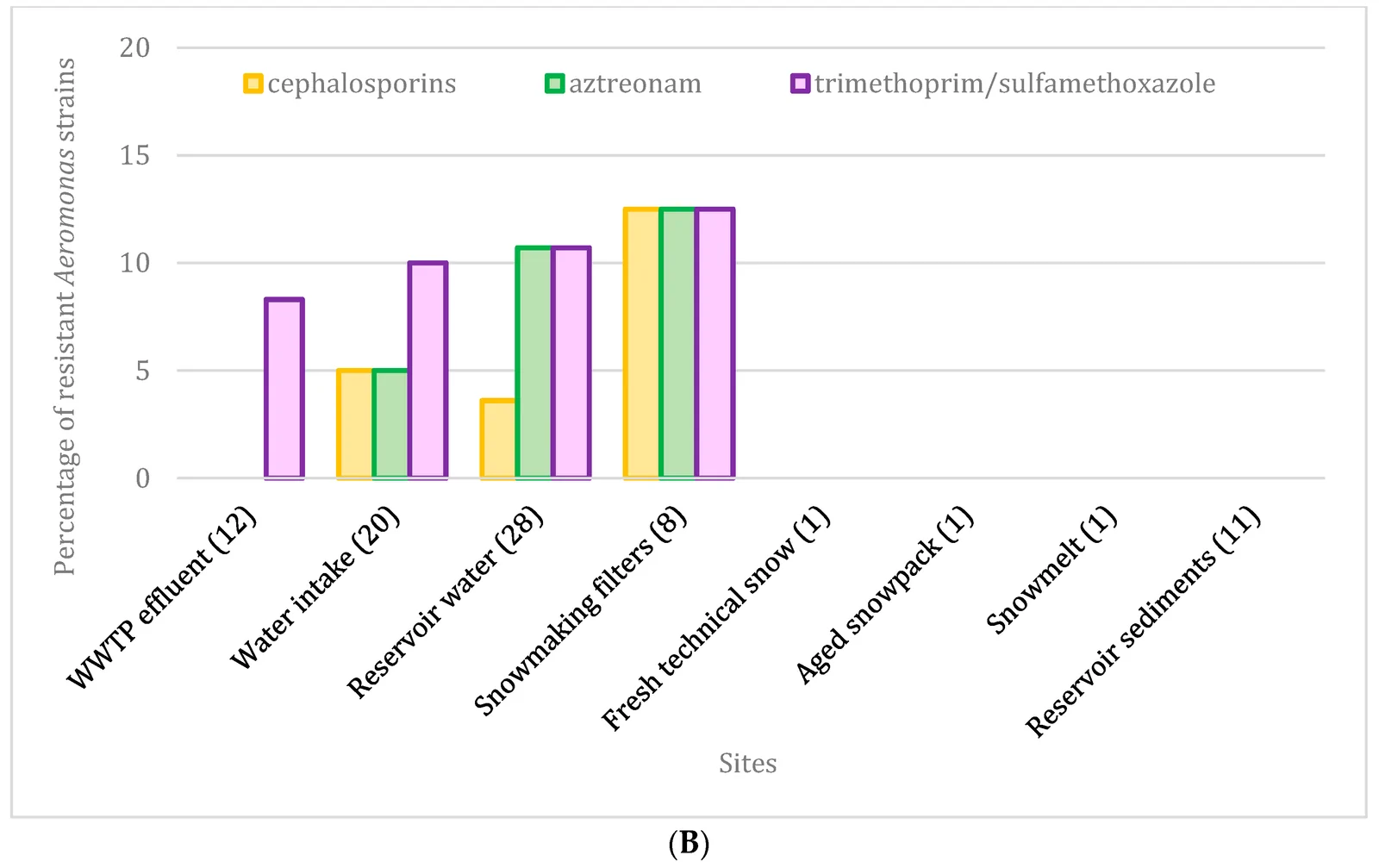
Percentage of antibiotic resistant and ESBL-positive *Enterobacteriaceae* (**A**) and *Aeromonas* (**B**) isolated along the snowmaking chain. Values in brackets, next to the site names show the numbers of isolated strains of each group shown in the figure. * indicates that the results should be treated with caution, given the small sample size of n = 2.

The proportions of phenotypically resistant strains and the variety of resistance phenotypes were lower in downstream compartments than in wastewater-influenced sites (Figure 5).

WWTP effluent contained the most complex resistance phenotype profile, with 83.3% of *Enterobacteriaceae* isolates showing ampicillin resistance, 50.0% amoxicillin/clavulanate resistance and 8.3% exhibiting third-generation cephalosporin (CTX) resistance. The trimethoprim/sulfamethoxazole (SXT) resistance phenotype was detected in 25% of *Enterobacteriaceae* and 8.3% of *Aeromonas* isolates. Water intake showed a similarly rich resistance profile in *Enterobacteriaceae*, but with reduced shares (drop to 18.2% for ampicillin, to 36.4% for amoxicillin/clavilanate and an undetected cephalosporin resistance). ESBL-positive *Enterobacteriaceae* comprised 33.3% of isolates at WWTP effluent, rising to 72.7% at the water intake. Ampicillin resistance in *Enterobacteriaceae* differed significantly among compartments (χ2 = 15.27, *p* = 0.0029), ESBL prevalence also differed significantly (χ2 = 19.11, 0 < 0.001) with its highest proportion in intake water (72.7%).

*Aeromonas* spp. became the dominant genus in intake water (20 of 31 isolates) and reservoir water (28 of 42 isolates) but Enterobacteriaceae retained the highest resistance burden (66.7% resistant to at least one antimicrobial, dominated by AMP resistance at 50.0% in reservoir water). *Aeromonas* resistance was comparatively low in all compartments where it was detected (Figure 5B). Snow cannon filters retained many resistant *Enterobacteriaceae* isolates (69.2% resistant to at least one antimicrobial agent, dominated by AMP resistance at 69.2% and AMC at 30.8%), while *Aeromonas* resistance still remained low (12.5%).

*Enterobacteriaceae* isolates from fresh and aged technical snow showed 100% and 66.7% resistance to at least one antibiotic, respectively (both results based on only n = 3, dominated by AMP resistance), with no or minor resistance among *Acinetobacter*, *Pseudomonas* and environmental bacilli/cocci in these compartments. *Enterobacteriaceae* from snowmelt water showed reduced resistance (28.6%, dominated by AMC). Reservoir sediments showed the lowest overall resistance of the entire chain.

### 2.5. ARG Richness of the Snowmaking System

**Figure 6:**
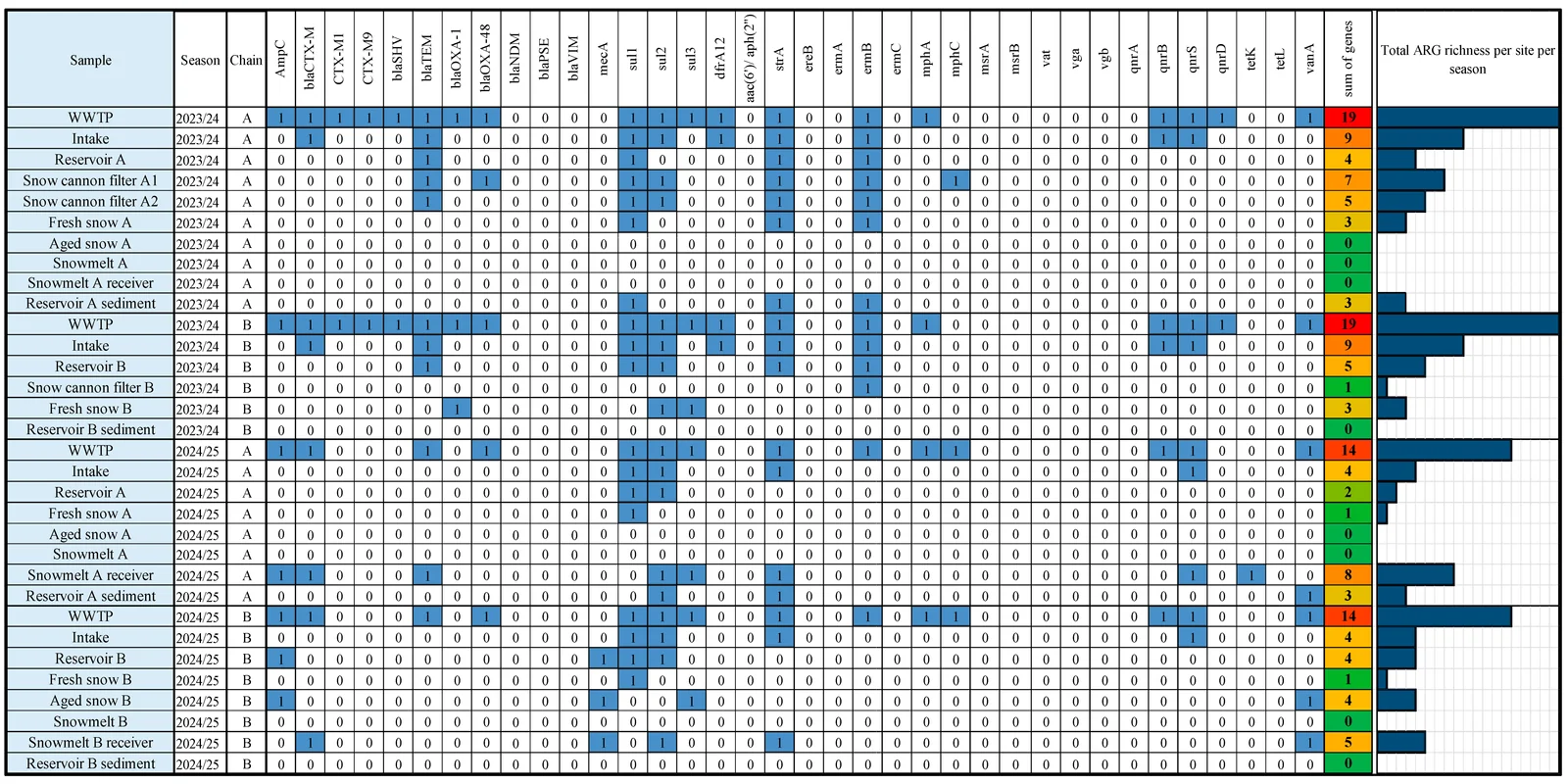
Presence of PCR products of expected sizes for the examined antibiotic resistance genes (ARGs) across environmental samples collected during two snowmaking seasons (2023/24 and 2024/25). Blue shading indicates gene presence (value = 1) in the gene-presence/absence matrix, while unshaded cells indicate absence (value = 0). In the “sum of genes” column, cell color reflects a red–yellow–green gradient scaled to ARG richness per sample, from red (highest richness) through yellow/orange (intermediate) to green (lowest richness, including zero). Samples include WWTP effluent, snow cannon filters, fresh and aged snow, snowmelt, and reservoir sediments from chains A and B. Shaded cells indicate detected ARGs; total ARG counts per sample are visualized alongside.

The gene detections reported below were based on PCR product size and were not confirmed by sequencing; they should therefore be considered presumptive (see Section 4.5). Antibiotic resistance gene (ARG) richness varied substantially between the snowmaking system compartments, with a clear reduction gradient from wastewater effluent to snowmelt and receiving waters (Figure 6).

WWTP effluent exhibited the highest mean ARG richness of all sampled compartments (mean = 16.5 genes per sample, Figure 6), harboring up to 19 target genes in the 2023/24 season. Water intake showed a clear dilution relative to WWTP effluent (mean 6.5; 9 genes in 2023/24 and 4 genes in 2024/25), retaining predominantly *sul1*, *sul2*, *strA* and *qnrS*. These two sites thus together represent the major source of ARGs in the snowmaking chain.

**Figure 7:**
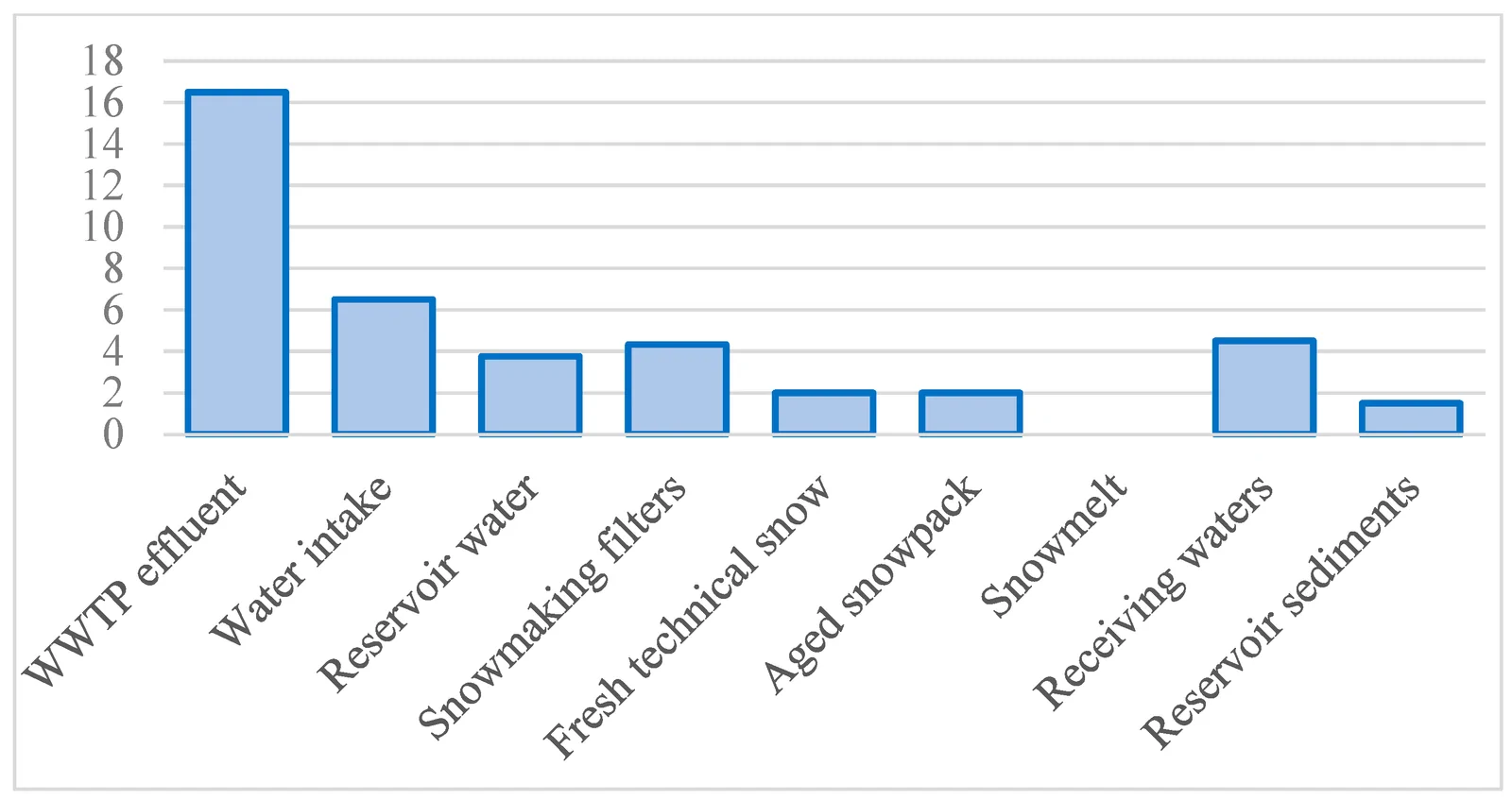
Mean ARG richness in the system compartments, averaged across both chains and seasons.

The ARG richness declined significantly along the technical snowmaking infrastructure. Reservoir waters showed intermediate richness values (from 2 to 5, mean 3.75 genes in reservoirs), retaining primarily sulfonamide (*sul1*, *sul2*) determinants in both seasons. Snow cannon filter samples in chain A yielded seven and five ARGs, exceeding the supplying reservoir water. This reflects the snow cannon filter role in blocking particulate matter. In this way at least a fraction of ARG and ARB is retained, reducing their spread in the wider environment (Figure 6 and Figure 7). In chain B, filter ARG richness (mean = 1) remained lower than in the chain A filters, the two chains are reported to differ qualitatively in filter design, which might be the cause of the observed differences.

Fresh technical snow was characterized by low but still detectable ARG richness in both seasons and both chains (mean = 2 genes), indicating that resistance determinants present in reservoir water were still detectable in fresh technical snow. The genes persisting in fresh snow were a reduced subset of those present in reservoir water, principally *sul1*, *strA*, and *ermB*, suggesting selective retention during snowmaking process. The *sul1* persisted in all fresh snow samples. Aged snowpack and meltwater represented the most effective reduction stages in the system. In both compartments ARG richness was zero in the majority of samples, consistent with clearly lower ARG detection in these compartments compared with earlier stages.

An interesting exception was observed in the snowmelt receiving waters in the 2024/25 season, when in chain A, the ARG richness reached 8, while in chain B it was 5. The value observed in chain A was the highest value recorded at any downstream site across the study sites. This clearly differed from the situation observed in 2023/24 (ARG richness = 0). The PCR products of the expected band sizes detected in this sample (*AmpC*, *blaCTX-M*, *blaTEM*, *sul2*, *sul3*, *qnrS*, *tetK*) included several not detected in the corresponding snowmelt, suggesting a possible runoff input or other processes rather than a direct technical snow-related source.

Finally, reservoir sediments in chain B were ARG-free, while in chain A in both seasons the ARG richness was 3. These PCR products comprised sulfonamide resistance determinants (*sul1* and *sul2*), streptomycin resistance determinant *strA*, erythromycin resistance determinant *ermB*, and *vanA*, which encodes the resistance to vancomycin.

### 2.6. Multivariate Integration: Identification of AMR Functional Zones

**Figure 8:**
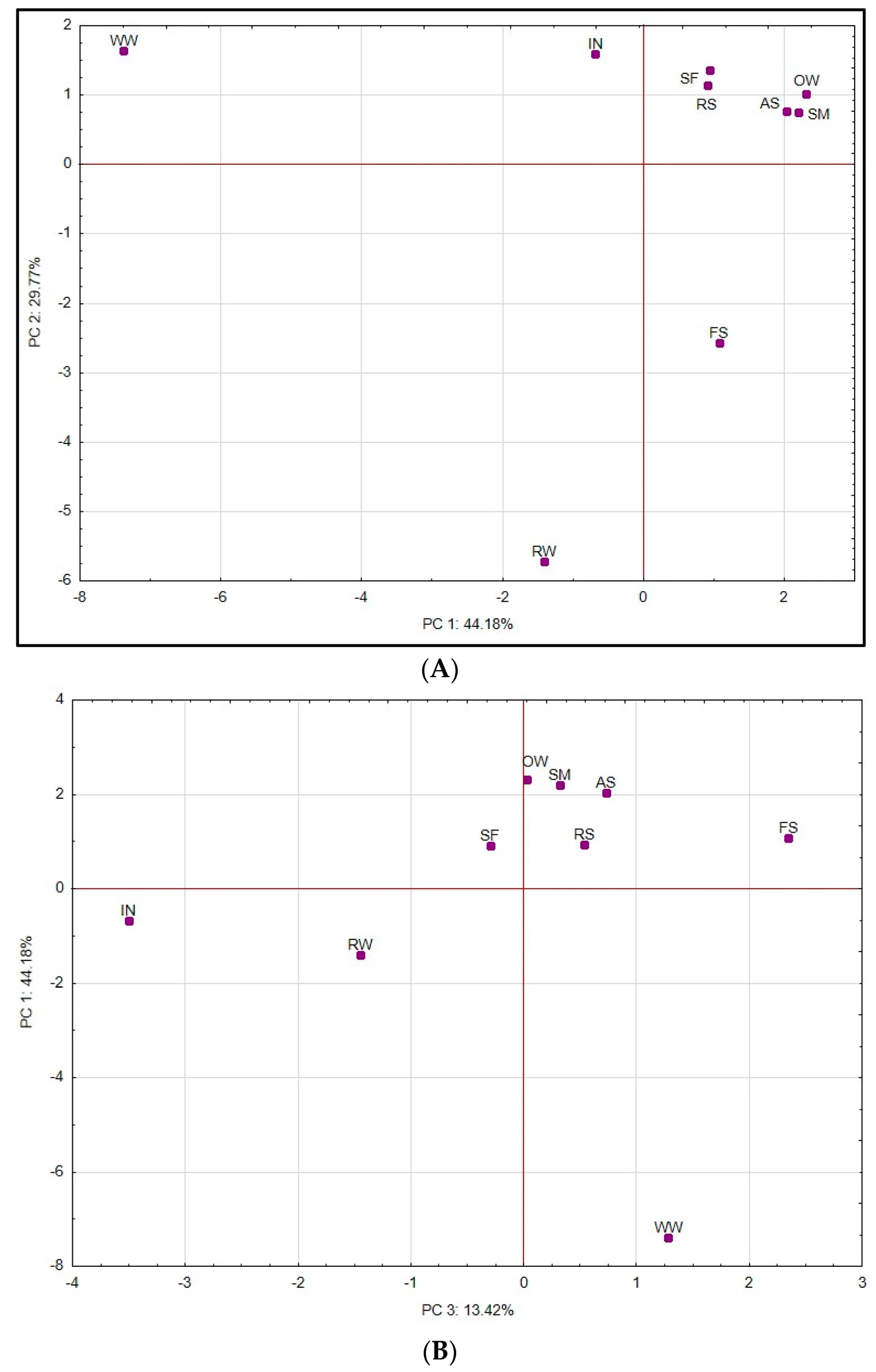

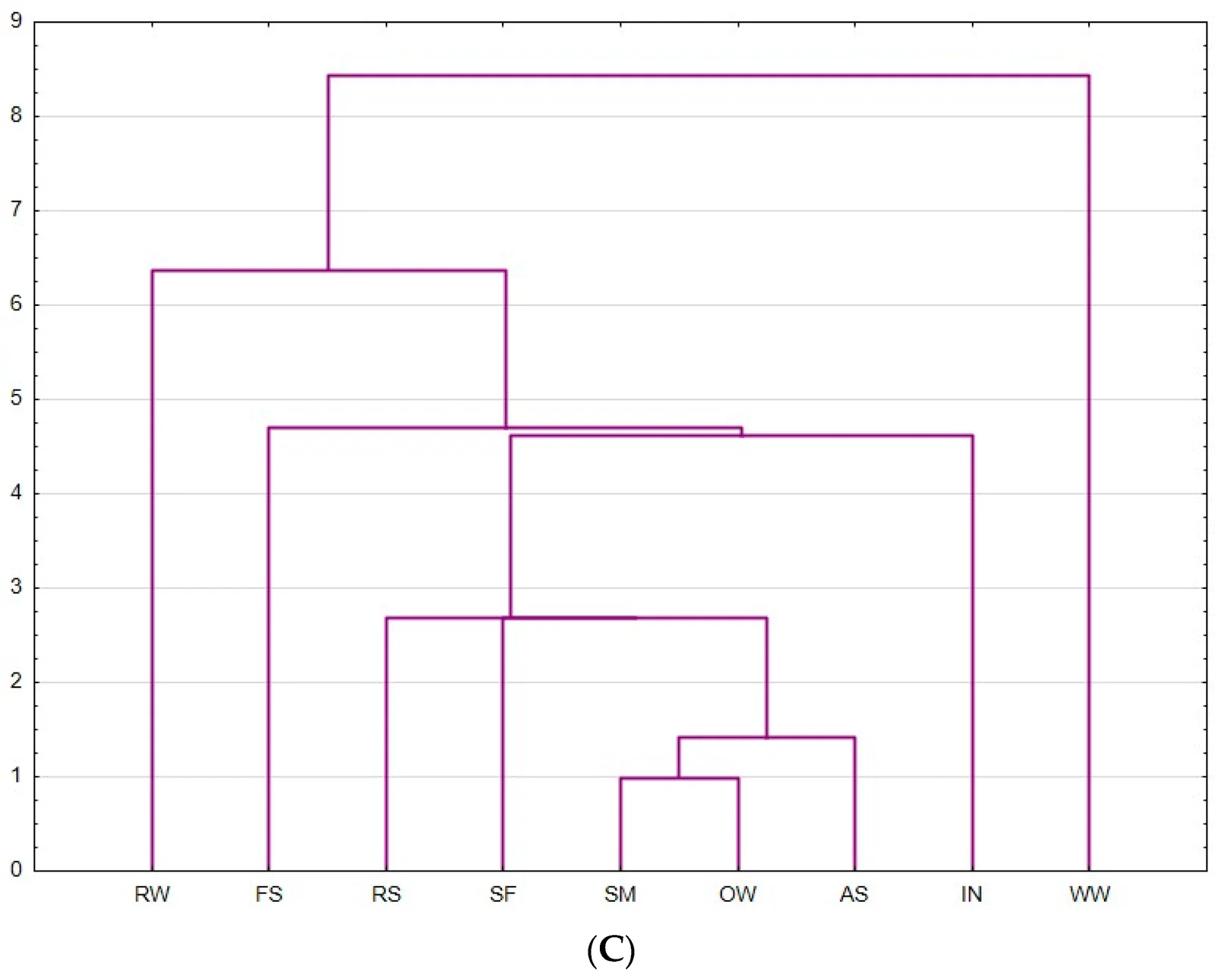
Multivariate analysis results: (**A**) PC1 vs. PC2 and (**B**) PC1 vs. PC3 PCA biplots. (**C**) Cluster analysis of the snowmaking system compartments, single linkage, Euclidean distance. Sites coded: WW = WWTP, IN = intake, RW = reservoir water, SF = snowmaking filters, FS = fresh snow, AS = aged snow, SM = snowmelt, OW = outflow/receiving stream, RS = reservoir sediments.

The PCA results of the simplified dataset revealed three principal components, explaining 87.37% of total variance (PC1 = 44.18%, PC2 = 29.77, PC3 = 13.42%) (Figure 8).

PC 1 was defined by eight variables (with factor loadings exceeding 0.70), mainly wastewater and AMR-associated: ARG richness (−0.964); *Enterobacteriaceae* SXT resistance (−0.909); *E. coli* counts (−0.914); and clindamycin (−0.942), trimethoprim (−0.950), sulfamethoxazole (−0.933), erythromycin (−0.887), and *E. faecalis* counts (−0.732). All loadings were negative, meaning PC1 represents a gradient from highly wastewater-affected samples (WW = wastewater effluent, IN = intake water, RW = reservoir water) to clean downstream compartments (SM = snowmelt water, AS = aged snow and OW = outflow water). PC1 can therefore be related to a wastewater-derived and antimicrobial load axis.

PC 2 was defined by five, solely antibiotic-associated, variables, i.e., ciprofloxacin (−0.991), enrofloxacin (−0.978), tylosin (−0.918), vancomycin (−0.858), and linezolid (−0.725). These antimicrobials were the ones found to accumulate in reservoirs in the chemical analysis Shannon diversity H-loaded positively on PC2 (+0.618), opposite to the antibiotic loadings, indicating that compartments with elevated antibiotic concentrations on this axis (i.e., reservoir water) also tended to have lower microbial diversity. PC2 can therefore be interpreted as associated with reservoir antibiotic enrichment.

PC3 was less clearly defined, with only ESBL prevalence exceeding the 0.70 threshold (−0.761); *Aeromonas* SXT resistance (−0.582) and the diversity indices H (−0.531) and Dm (−0.468) contributed moderately. This axis broadly separated ESBL prevalence and microbial diversity from the antibiotic-associated variables, but should be interpreted with caution given the explained comparatively low variance and weaker loadings.

The location of study sites on PC1 and PC2 designated four functional zones within the snowmaking infrastructure (Figure 8A). WWTP effluent was strongly isolated at the negative extreme of PC1. Reservoir water was the clearest outlier on PC2, positioned orthogonally to other sites at its negative extreme, distinct from intake water despite both being wastewater-influenced. Snowmaking filters and reservoir sediment formed a loosely transitional cluster near the origin, while aged snow, snowmelt, and outflow water grouped together at the positive extreme of PC1. Fresh snow occupied a distinct position, separate from both the wastewater-influenced sites and the aged-snow/outflow group.

Cluster analysis of the nine compartments (Figure 8B) distinguished WWTP effluent and intake water as the two most distinct compartments, each separating from the rest of the system at the highest distances. Reservoir water and fresh snow formed a distinct pair, separate from a tighter cluster comprising snow cannon filters, reservoir sediment, aged snow, snowmelt and outflow water—within which snowmelt and outflow water were the most closely related pair.

## 3. Discussion

### 3.1. Wastewater Effluent as Primary Significant AMR Source

The WWTP effluent represented a major sampled source of antibiotic residues, ARB and ARGs in the snowmaking system, consistent with its known role as a principal point source of AMR determinants in aquatic environments [3,18,19] and our earlier, dedicated studies of this specific treatment plant and its surrounding catchment [12,20,21]. Apart from the highest counts of bacterial indicators of water quality, this site was characterized by the presence of antibiotics from 6–7 classes, a high prevalence of ARB, ESBL-positive *Enterobacteriaceae* strains (33.3%) and high ARG richness. Co-occurrence of these micropollutants indicates that the conventional mechanical–biological treatment is insufficient to eliminate pharmaceutical compounds. On one hand, this situation may exert selection pressure (sub-lethal antibiotic concentrations) on the environmental bacterial community and, on the other hand, provide readily available material for this drug resistance to be developed (from ARG carried by discharged bacteria), as has been reported in WWTPs throughout Europe [3,18,22]. We note that the current single-point sampling design does not, on its own, distinguish WWTP-derived inputs from upstream, diffuse or local sources reaching the same stream section; this distinction is instead supported by the influent–effluent and multivariate evidence reported in our earlier work in this catchment.

The approximately 23-fold reduction in *E. coli* between WWTP effluent and intake water (22,422 to 967 CFU/100 mL) suggests effective dilution and natural attenuation during river flow. However, this was not reflected by proportional ARG reduction (61% decrease from mean 16.5 to 6.5 genes) or antibiotic concentration decrease. Such a divergence between biological and chemical/molecular contaminant reduction rates suggests that intake water that enters the snowmaking infrastructure cannot be assumed to be devoid of AMR, even when fecal indicator counts are relatively low.

### 3.2. The Reservoir Paradox in AMR Determinants: Simultaneous Decline in ARB/ARGs and Accumulation of Antimicrobials

The most scientifically novel finding of this study is the diverse fate of various categories of AMR determinants within storage reservoirs. While bacterial indicators and ARG richness declined from intake to reservoir water, concentrations of several antibiotic compounds substantially exceeded intake levels and, in some cases, surpassed even the WWTP effluent concentrations. This pattern was followed by fluoroquinolones (ciprofloxacin, enrofloxacin and ofloxacin all reaching normalized intensity 1.0 in reservoir B in 2023/24), tylosin and even vancomycin.

Such observation may be explained by a few co-existing mechanisms. Fluoroquinolones and macrolides (i.e., tylosin and erythromycin also detected through the cycle) can adsorb onto solid particles which makes it difficult to understand their fate in the environment, but it can also explain their increased concentration in the reservoirs [23]. In flowing river water this fraction is transported downstream, but in a static reservoir it accumulates and can be resuspended cyclically, which is reflected in the still detectable concentrations of these antimicrobials in aged snow, snowmelt water and reservoir outflow. The hydraulic residence time of the reservoirs, ranging from days to weeks during the snowmaking season, allows for the accumulation of more stable compounds, such as fluoroquinolone and macrolide antibiotics [10,24]. The detection of tylosin, which reached the maximum normalized concentration in reservoir A and close to maximum in reservoir B is particularly noteworthy. Tylosin is a macrolide antibiotic used only in veterinary medicine and its high concentration in reservoirs, without being detected in WWTP effluent, suggests its non-point sources, e.g., agricultural runoff or atmospheric deposition from surrounding farmed areas [25,26]. Similarly, vancomycin detection in both reservoirs in 2023/24 season most likely reflects its environmental persistence [27] which could allow gradual accumulation from trace input, though this was not directly tracked over time in this study design. Together, these two cases illustrate that unexpected antibiotic patterns in the reservoirs may arise from a combination of non-point sources (agricultural runoff, atmospheric deposition) and the environmental persistence of certain compound classes rather than from a single, easily identifiable input. Additionally, these findings indicate that water storage in retention reservoirs may accumulate antibiotics of various classes from point and non-point sources and that this process may completely avoid standard monitoring.

The shift in dominant bacterial composition from *Enterobacteriaceae* at the intake to *Aeromonas* spp. in reservoir water (27 of 46 isolates; 59%) is another observation that demonstrates the specific conditions of reservoirs. *Aeromonas* spp. are well-adapted to oligotrophic, cold, static conditions, which gives them advantage over mesophilic *Enterobacteriaceae* under longer residence times of stored water [28,29]. The reduction of ESBL-positive *Enterobacteriaceae* from 72.7% at intake water to 50% in reservoirs, and their further disappearance downstream, coincided with a shift towards environmental taxa. This pattern is consistent with, but not direct evidence of, altered selective conditions in reservoirs favoring environmental over clinically important taxa; distinguishing selection from simple dilution or differential survival would require additional, targeted experiments. The significant positive correlation between antibiotic concentration and ARG richness, with the lack of correlation between antibiotic concentration and phenotypic resistance, indicates that antibiotic pressure in this system is statistically associated with ARG accumulation but not directly with phenotypic resistance levels.

### 3.3. Snow Cannon Filters as Infrastructure Elements That Affect the Fate of AMR

ARG richness in chain A filters (7 and 5 genes) exceeded that observed in its supplying reservoir (3–5 genes) and harbored the highest share of AMP-resistant *Enterobacteriaceae* out of the examined infrastructure components (69.2%), while *Aeromonas* resistance remained comparatively low (12.5%). Microbial community diversity was reduced compared with the reservoir (H = 1.415/1.288 vs. 1.402) with elevated dominance (λ = 0.113–0.117 vs. 0.073), suggesting selective retention of some bacterial groups in filter mesh/surfaces under chronic hydraulic stress. Material collected from the chain B filter harbored only one ARG, no culturable bacteria and the community diversity as well as dominance remained similar as at the intake (H =1.831 vs. 1.833; λ = 0.026 vs. 0.033).

Snow cannons are identical across both chains and operate under the same water supply pressure; however, the filter units themselves differ between chain A and chain B, but neither pore size nor operating pressure is documented by the manufacturer or the resort operator for either filter type. We therefore report the marked difference in ARG richness and microbial diversity between chain A and chain B filter material as a qualitative, site-specific observation. Controlled comparisons across filters with documented, contrasting specifications would be needed to test this hypothesis directly. The elevated ARG richness in the material collected from the chain A filter compared with the reservoir water may reflect either concentration of ARG-bearing bacteria or the in situ horizontal gene transfer within the material collected on filter pores. The detection of *blaOXA-48* carbapenemase gene in chain A filter material in 2023/24 underscores the relevance of filters in ARG trapping, including genes determining the resistance to last-resort carbapenem antibiotics [30]. The elevated prevalence of ARG richness and AMP-resistant *Enterobacteriaceae* indicate that snow cannon filters may retain and accumulate bacterial resistance determinants [19].

### 3.4. Technical Snow as an AMR Reducing Environment

Technical snow and its ageing process appeared to be the most effective AMR reduction pathway in the system. Aged technical snow showed lower antibiotic concentrations, ARG detection rates and viable ARB counts compared with earlier system compartments. The overall decrease of *E. coli* counts from WWTP to receiving stream, with major contribution to this reduction by technical snowmaking, indicates that this process may represent an important step in pollutant and micropollutant attenuation.

The observed micropollutant reduction is driven by a few mechanisms. First, elimination of antimicrobials is mainly caused by their photodegradation and hydrolysis, which are major pathways of antibiotic degradation in aquatic and semi-aquatic environments [10]. As for microorganisms, including the resistant ones, phase transition from liquid to ice causes severe physical stress, i.e., crystal ice formation, osmotic disruption or mechanical damage, explaining the decrease in their numbers [31].

However, enrofloxacin and trimethoprim partially escaped the elimination processes during snow ageing and snowmelt, as both these compounds were detected in snowmelt water in low but quantifiable concentrations. Enrofloxacin is a very widely used veterinary fluoroquinolone antibiotic, the persistence of which in aqueous environment is very high [32]. Also, trimethoprim in combination with sulfamethoxazole is one of the most frequently prescribed antimicrobial in human medicine and is also largely stable in aquatic conditions [33].

The PCR product corresponding to the *sul1* gene band size was detected in all fresh snow samples, consistent with its environmental persistence. It confers resistance to sulfonamides and is commonly carried on class 1 integrons, which can be either chromosomally or plasmid-borne; *sul1* is among the most ubiquitous ARGs in natural aquatic environments globally, and is suggested as an indicator gene (along with *blaTEM* and *intl1*) of contamination to assess the state of antimicrobial resistance in the environment [34,35]. Its presence in the fresh snow does not necessarily indicate viable *sul1*-bearing bacteria, but still their extracellular DNA can be present in snow and can be transferred horizontally to other bacteria.

### 3.5. Reservoir Sediments as ARG/Biodiversity Accumulation Niches

Reservoir sediments were characterized by increased diversity (H = 1.592–1.688; observed OTU richness 169–243), the presence of culturable indicators (*E. coli* counts of 160 CFU/g and *E. faecalis* 1330 CFU/g) and ARG presence in chain A (*sul1*, *sul2*, *strA*, *ermB*, *vanA*), while the phenotypic resistance among sediment isolates was the lowest among all examined compartments, suggesting that ARGs’ presence and persistence in sediments does not necessarily translate into elevated culturable resistant phenotypes. High biodiversity indices coupled with ARG detection exceeding the one observed in upstream sites is consistent with the role of reservoir sediments as long-term sources of ARB and ARGs due to favorable conditions prevailing therein [26,36,37]. Antibiotic residues were not chemically quantified in sediment in this study; their presence or absence in this matrix therefore remains to be determined, and the elevated ARG richness observed here may reflect historical exposure, in situ horizontal gene transfer, or ARG persistence independent of current antimicrobial pressure. Accumulated organic matter, anoxic microenvironments, protection from UV light, prolonged residence time each promote biofilm formation on solid particle surfaces and as such provide protective conditions for ARB [26]. With these factors, sediments provide favorable environments for horizontal gene transfer for microbial populations within reservoirs. Human-mediated activities, such as sediment removal during reservoir cleaning/desilting may contribute to the further dissemination of ARB and ARGs [36].

The detection of a *vanA*-sized PCR product in the reservoir sediment (apart from WWTP, aged snow and snowmelt receiver) is a noteworthy observation of this study. However, with no positive-control strain available for this gene and amplicon identity not confirmed by sequencing, this finding should be interpreted with caution. Coupled with a similarly presumptive *blaOXA-48*-sized product in WWTP and snow cannon filter material, there is the tentative suggestion that snowmaking systems may catch and harbor genetic determinants of resistance to last resort antibiotics. Sequence-level confirmation is needed before drawing firmer conclusions about their clinical significance.

### 3.6. Four Functional Zones and Their Roles as Barriers and Vectors

Multivariate analyses (PCA and CA) were conducted to identify the major patterns that dominated the examined system. These analyses suggested the following exploratory four-zone grouping of the snowmaking chain, based on compartment-level averages: (I) sites contaminated with wastewater-associated AMR determinants (WWTP effluent, intake water); (II) reservoir water characterized with elevated concentrations of antibiotics and reduced biological AMR indicators; (III) contaminant transition and attenuation zone (snow cannon filters, fresh and aged snow, snowmelt, outflow/receiving stream), where chemical, molecular and phenotypic AMR indicators were consistently lower than in the wastewater-influenced compartments; and (IV) reservoir sediments, that contain the remnants of contamination like indicator bacteria, including resistant strains (Figure 8).

Snow cannon filters grouped with the downstream AMR reduction zone (reservoir sediment, aged snow, snowmelt and outflow water), on the opposite side of PC1 from the wastewater-impacted sites. This position indicates that filters can act as one of the retention steps along the snowmaking chain, consistent with the progressive contaminant removal, which follows the initial reduction achieved at the water intake and then in the reservoirs. The practical implication of this finding is that filter maintenance, including regular replacement and cleaning, appears to be an effective and worthwhile practice. Contaminants that pass through the intake and reservoir (including sedimentation or UV light disinfection) are further retained at the filter mesh, which results in technical snow cleaner than the water used for its production.

The answer to the question of whether snowmaking infrastructure acts as an AMR barrier or a vector is that it can act both ways. This depends on the individual AMR category and infrastructure component and on the way that it is operated. Importantly, fecal contamination indicators were up to four orders of magnitude lower, with antibiotic concentrations also lower and ARG detection reduced, in aged technical snow compared with WWTP effluent. On the other hand, the reservoirs accumulate some antibiotic classes above their input levels and the fine-pore filters accumulate ARGs. On the other, fresh snow deposits viable resistant bacteria and multiple antibiotic residues onto the ski slopes used by hundreds of thousands of people, while reservoir sediments accumulate resistance determinants that can be transported further during reservoir dredging.

### 3.7. One Health Implications and Management Recommendations

From a One Health perspective, ski resorts represent unique AMR risk environments that combine seasonally changing numbers of tourists, water reuse via snowmaking, direct recreational contact of people with technically produced snow and post-season snowmelt distribution of accumulated contaminants further downstream. The current study is, to our knowledge, the first to document the full AMR burden of this exposure pathway and to identify the specific infrastructure components through which it operates. The detection of *vanA*-sized PCR product in sediments and *blaOXA-48*-sized PCR product in filter material required sequencing-based confirmation, suggesting that snowmaking systems might be considered in environmental AMR risk assessments. This is particularly important as technical snowmaking becomes an indispensable element of most ski resorts in the northern hemisphere and this process often uses water from rivers and streams affected by WWTP effluents [15,17].

Several management recommendations follow from our findings. Water quality monitoring at ski resorts using river water for snowmaking should incorporate ARG screening and antibiotic residue analysis, particularly for intake and reservoir water. We found an unexpectedly high share of ESBL-producing *Enterobacteriaceae* at the intake (72.7% of isolates, compared with 33.3% in the WWTP effluent), suggesting that the proximity of the treatment plant or fecal indicator counts alone are not representative enough for AMR risk assessment in some system compartments. Filter performance also differed between the two studied chains, suggesting that filter design and maintenance schedule may affect how much AMR is retained. Resorts operating various filter types may benefit from comparing filter performance within their infrastructure. Nevertheless, snow cannon filters would also benefit from regular decontamination during the snowmaking season to prevent biofilm accumulation. In the case of ski resorts drawing water from surface sources, AMR risk assessment is a reasonable step when planning new snowmaking infrastructure and advanced pre-treatment may be worth considering at resorts drawing water directly from WWTP-impacted streams, as the conventional treatment examined in our study site did not prevent antibiotic accumulation in reservoir water. As this study did not assess exposure magnitude, dose–response relationships, or actual infection risk to workers or recreational users, the above should be read as reasonable precautions suggested by our findings, rather than risk levels demonstrated here.

### 3.8. Limitations

Several limitations should be acknowledged. The single-resort design limits direct generalization and comparative studies in different resort types (with and without storage reservoirs, with different filter designs and at different altitudes) are needed to establish the general patterns described in this study. Because multiple isolates could be recovered from the same environmental sample, isolate-level resistance percentages reported here should be interpreted as descriptive of the cultivable resistant fraction recovered per sample, rather than as statistically independent observations; environmental-level replication is provided by the multiple sampling dates and duplicate chains rather than by isolate counts. The targeted 37-gene PCR panel cannot capture the full resistome, and future work should complement qualitative screening with quantitative PCR for gene copy number and with metagenomic approaches, particularly for sediment and filter biofilm compartments.

## 4. Materials and Methods

### 4.1. Study Area and Sampling Design

The study was conducted over two consecutive winter seasons (2023/24 and 2024/25) at a major, high-capacity ski resort located in southern Poland (western Carpathians), situated at an altitude of approximately 650–910 m a.s.l. The selected facility is one of the largest and most intensively operated ski complexes in Poland, characterized by high tourist traffic during the winter season. The resort features an extensive infrastructure, including over a dozen chairlifts and ski lifts, and relies heavily on an advanced, intensive artificial snowmaking system supplied by local surface water resources. The mountain stream supplying intake water for snowmaking receives treated effluent from a municipal wastewater treatment plant (WWTP), which serves the local community, operates with a designed capacity of c.a. 800 population equivalents and employs mechanical–biological treatment. The distance between the WWTP effluent receiver and the water intake point for snowmaking is approx. 2.5 km.

**Figure 9:**
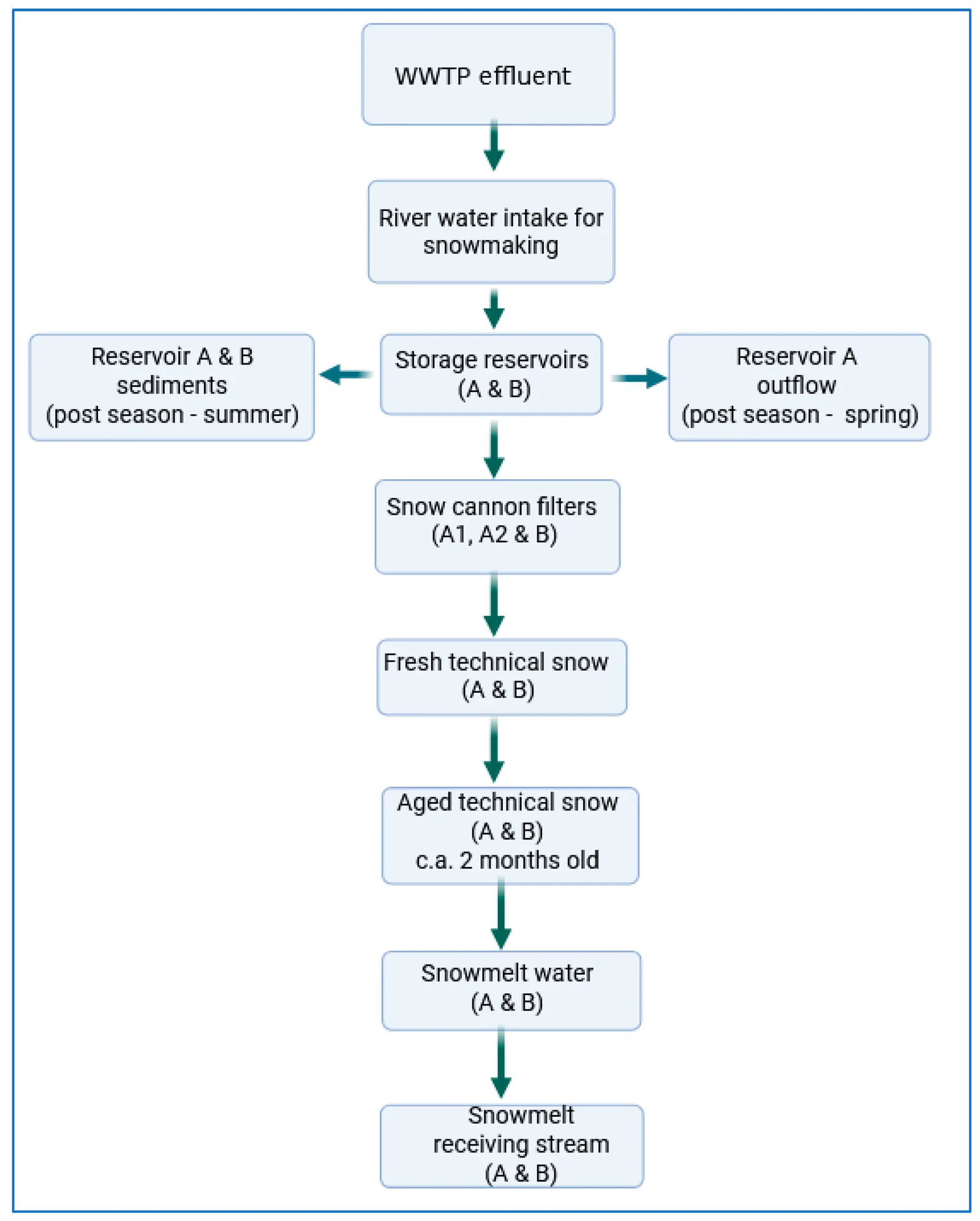
Layout of the sampling points.

The resort operates two independent snowmaking chains (chain A and chain B), each consisting of (i) a water intake point on the mountain stream; (ii) storage reservoirs equipped with aeration and UV light-based disinfection; (iii) snow cannon filters; (iv) snow cannons producing technical snow deposited on designated ski slopes; and (v) a receiving stream collecting meltwater outflow. The two chains differ in their snow cannon filter design (chain A employs a finer-pore filtration system, chain B a coarser one). Sampling points included each transformation stage along both chains: WWTP effluent (shared source), water intake (shared), water of reservoir A and reservoir B, snow cannon filters (filter A1, filter A2, filter B), freshly produced technical snow (fresh snow A and B), aged snow after approximately 8 weeks of deposition (aged snow A and B), snowmelt (snowmelt A and B), meltwater-receiving stream (receiver A and B), post-season reservoir outflow (outflow A), and post-season reservoir sediments (sediment A and B). The spatial layout of sampling points is shown in Figure 9.

In each season, three replicate samples were collected simultaneously at each sampling point during campaigns coinciding with peak snowmaking operation (December–February). The samples were collected in a total of 12 sampling campaigns (exact and generalized dates given in Supplementary Table S1). Not every analysis was performed on every sampling date (Supplementary Table S1 provides a detailed list of analyses conducted for individual samples on each date). Where compartment-level values are reported as a single number in the main text, these represent the mean of all available sampling dates for that compartment and chain, not repeated measurements of the same water parcel moving through the system. Because compartments were not sampled as the same moving parcel of water, and given variable and largely unmeasured transit and residence times along the chain, terms such as “removal”, “accumulation”, “elimination” and “reduction” describe the differences in concentration or count between compartments, not the measured process rates or mass-balance outcomes.

### 4.2. Sample Collection and Preservation

All samples were collected under sterile conditions using autoclaved or single-use equipment. Water samples (WWTP effluent, intake, reservoir water, snowmelt, receiving stream water) were collected in 1000 mL sterile wide-neck polypropylene bottles pre-rinsed three times with sample water. Snow samples were collected by first removing the top 2 cm layer, then extracting a core using a 1.0 m × 10 cm sterile polycarbonate snow corer; snow cores were placed in sterile polypropylene string bags and melted. Sediment samples (~50 g wet weight) were collected from the deepest accessible point of each reservoir using a sterile stainless-steel scoop into sterile 100 mL polypropylene containers. Snow cannon filter material was sampled by swabbing the internal filter surfaces with sterile swabs. The samples were collected with two swabs per filter, each collected at the inlet of the filter, on a surface area approx. 10 cm^2^. Swabbing was performed onsite, within one hour of the filter removal from the snow cannon. The filters are exchanged once per 24 h of continuous operation. After being transported to the laboratory, the samples were processed immediately. Aliquots for molecular and chemical analyses were stored at −20 °C for a maximum of four weeks. Swabs were eluted in 1 mL of sterile 0.85% saline solution by vortexing for 1 min, and the eluate was used directly for culture and molecular analyzes.

### 4.3. Chemical Analysis of Antimicrobial Agents

Twenty-one antimicrobial compounds from 14 pharmacological classes were targeted: aminopenicillins (amoxicillin, ampicillin); 2nd generation cephalosporins (cefoxitin); 3rd generation cephalosporins (ceftazidime); fluoroquinolones (ciprofloxacin, enrofloxacin, ofloxacin); lincosamides (clindamycin); tetracyclines (doxycycline, oxytetracycline, tetracycline); macrolides (erythromycin, tylosin); aminoglycosides (gentamicin, netilmicin); oxazolidinones (linezolid); carbapenems (meropenem); semi-synthetic penicillins (piperacillin); sulfonamides (sulfamethoxazole); dihydrofolate reductase inhibitors (trimethoprim); and glycopeptides (vancomycin).

Antimicrobial agents were extracted by solid-phase extraction (SPE) using Oasis HLB cartridges (500 mg sorbent, 6 cc; Waters, Milford, CT, USA) following the procedure described by Stankiewicz et al. [16]. Briefly, 500 mL water or snowmelt samples were passed through conditioned HLB cartridges, eluted with methanol, evaporated to dryness under an air stream, and reconstituted in 1 mL of 10% methanol in ultrapure water. Chemical analysis of antimicrobial residues was performed on water samples (wastewater effluent, intake water, reservoir water, snowmelt water and receiving stream water) and snow samples (fresh technical snow, aged snowpack); filter material and sediment samples were not subjected to chemical analysis and are therefore not represented in the antibiotic concentration dataset.

Concentrations of antimicrobials were quantified by UHPLC-MS/MS using an Agilent 1290 Infinity system coupled with an Agilent 6460 Triple Quad mass spectrometer (Santa Clara, CA, USA) in positive and negative electrospray ionization mode. Two diagnostic precursor-to-product ion transitions were monitored per compound, as described by Lenart-Boroń et al. [12]. Limits of detection (LODs) ranged from 0.11 to 936.75 ng/L and limits of quantification (LOQs) from 0.33 to 2838.65 ng/L; SPE recovery ranged from 9.94% to approximately 100% depending on the compound [20]. Detailed values of LOD, LOQ, and SPE recovery values are shown in Supplementary Table S1. A concentration of “0” indicates a result below the LOD, while “+” indicates that the signal was detected, but fell between LOD and LOQ and could not be reliably quantified. Numeric values represent quantified concentrations at or higher than LOQ and corrected for compound-specific SPE recovery.

### 4.4. Microbiological Analyses

General microbiological quality was assessed by standard culture-based methods. Water and snowmelt were analyzed by pour-plate (1 mL) and membrane filtration (100 mL) methods. Sediments were serially diluted (1 g in 9 mL sterile physiological saline) before plating. For filter-swab eluates, 100 μL was surface plated. Selective media were used: TBX agar for *Escherichia coli* (turquoise colonies; 44 °C, 24 h); Slanetz–Bartley agar for *Enterococcus faecalis*/*E. faecium* (36 ± 1 °C, 72 h); Baird–Parker agar for coagulase-positive staphylococci (36 ± 1 °C, 48 h); and SS agar for *Salmonella* spp. (36 ± 1 °C, 24 h). All media were obtained from Biomaxima (Lublin, Poland). Results are expressed as CFU/100 mL (membrane filtration), CFU/mL (pour plate of water and swab eluate) or CFU/g wet weight (sediments). For the isolation and identification of clinically relevant organisms, Brilliance UTI Clarity Agar (Thermo Fisher Scientific, Waltham, MA, USA) was used (culture at 36 ± 1 °C, 24 h). This is a chromogenic medium on which colony color reflects species- or genus-specific enzymatic activity. Colony selection was based on chromogenic differentiation of colonies. For each plate, one representative colony of each visually distinct color/morphotype (e.g., pink, blue, turquoise, purple, pink with a halo, white, colorless etc.) was subcultured and identified to the species level by MALDI-TOF MS (Bruker Daltonics, Bremen, Germany; MBT Compass library v. 4.1), in order to capture the phenotypic and taxonomic diversity present on each plate while limiting redundant sampling of colonies with identical, already-represented phenotypes. Because chromogenic color on this medium does not correspond one-to-one with genus, several colonies selected as visually distinct morphotypes were subsequently identified by MALDI-TOF MS as belonging to the same genus, mostly *Aeromonas* spp. and *Enterobacteriaceae* genera, which produced colonies of variable color and morphology on this medium. This chromogenic variability accounts for the comparatively high number of Aeromonas and Enterobacteriaceae isolates recovered per sample. No molecular typing (e.g., rep-PCR) was performed to confirm clonal independence among same-species isolates recovered from the same plate.

Antimicrobial susceptibility testing was performed by disc diffusion on Mueller–Hinton II agar (Biomaxima), following current EUCAST guidelines [38]. Twenty-one antimicrobial agents from eight classes were tested: aminoglycosides (amikacin 30 μg, gentamicin 10 μg, tobramycin 10 μg); beta-lactams, including penicillins (ampicillin 10 μg, amoxicillin/clavulanic acid 20/10 μg, piperacillin/tazobactam 100/10 μg), cephalosporins (ceftazidime 30 μg, cefotaxime 30 μg, cefoxitin 30 μg, cefepime 30 μg), monobactams (aztreonam 30 μg), and carbapenems (meropenem 10 μg); fluoroquinolones (ciprofloxacin 5 μg, enrofloxacin 5 μg, levofloxacin 5 μg); macrolides (erythromycin 15 μg, tylosin 15 μg); lincosamides (clindamycin 2 μg); folate pathway inhibitors (trimethoprim/sulfamethoxazole 1.25/23.75 μg); tetracyclines (tetracycline 30 μg); and glycopeptides (vancomycin 30 μg). The discs were obtained from Oxoid (Basingstoke, UK). Growth inhibition zone diameters were measured after 18–24 h of culture at 36 ± 1 °C and interpreted according to the EUCAST breakpoint values obtained from tables valid at the time of examination: version 13.1 for isolates collected in 2023, version 14.0 for isolates collected in 2024 and version 15.0 for isolates collected in 2025 [38]. Isolates categorized as “susceptible, increased exposure” (I) were grouped with resistant isolates for the purpose of reporting reduced susceptibility, in accordance with current EUCAST terminology. Enrofloxacin has no EUCAST clinical breakpoint, as it is not licensed for human use and current veterinary guidance recommendations have withdrawn their own enrofloxacin breakpoints for *Enterobacteriaceae*. Enrofloxacin results are therefore reported throughout as inhibition-zone diameters (mm) without categorical interpretation, for every organism group tested. ESBL production in *Enterobacterales* was confirmed by the double-disc synergy test [39]. Inducible clindamycin resistance in staphylococci was assessed by the D-zone test [40]. Quality control strains *E. coli* ATCC 25922, *P. aeruginosa* ATCC 27853 and *S. aureus* ATCC 29213 were used as controls.

### 4.5. Molecular Analyses

Total environmental DNA was extracted from water (500 mL), snowmelt water (500 mL), snow cannon filter swabs (1 mL eluate) and sediment (0.5 g wet weight). Water and snowmelt water samples were filtered through 0.22 μm sterile cellulose nitrate filters (BioSpace, Warsaw, Poland). Filters were placed in 60 mm Petri dishes, covered with 1 mL sterile 0.85% NaCl and shaken at 120 rpm overnight. The resulting suspension combined with filter-surface swab eluates was centrifuged at 10,000× *g* for 10 min; the pellet was resuspended in 200 μL Tris buffer. DNA was extracted from all sample types using the Genomic Mini AX Bacteria + Spin kit (A&A Biotechnology, Gdansk, Poland) according to the manufacturer’s instructions.

Thirty-seven genetic determinants of antimicrobial resistance were screened by conventional PCR, targeting genes conferring resistance to aminoglycosides (*aac(6’/aph(2’)*); cephalosporins (*AmpC*) and beta-lactams (with beta-lactamase-encoding *blaPSE* and extended-spectrum beta-lactamase-encoding *blaTEM*, *blaSHV*, *blaCTX-M*, *blaCTX-M-1*, *blaCTX-M-9*, *blaOXA-1*); carbapenemase-encoding *blaNDM*, *blaOXA-48*, and *blaVIM*; fluoroquinolone resistance (*qnrA*, *qnrB*, *qnrD* and *qnrS*), macrolide-lincosamide-streptogramin B (MLSb) and resistance (*ereB*, *erm*A, *erm*B, *erm*C, *msrA*, *msrB*, *mph*A, *mphC*, *vatA*, *vatB*, *vga* and *vgb*); methicillin resistance (*mecA*); streptomycin resistance (*strA*) and sulfonamide resistance (*sul1*, *sul2*, *sul3*); tetracycline resistance (*tetK*, *tetL*); trimethoprim resistance (*dfrA12*); and vancomycin resistance (*vanA*). Primer sequences, annealing temperatures and amplicon sizes are provided in Supplementary Table S2. PCR reactions were performed in 25 μL volumes containing 50 ng template DNA, 12.5 pmol of each primer, 2.0 mM dNTPs, 1× PCR buffer and 2.4 U Taq polymerase (PCR Mix Plus Green; A&A Biotechnology, Gdansk, Poland) in a T100 thermal cycler (Bio-Rad, Hercules, CA, USA). Each PCR included a no-template control (reaction mixture without DNA template) and DNA isolates from bacterial strains confirmed as ESBL-positive *E. coli*, MRSA (*mecA*-positive) or MLSb-positive staphylococci. No positive control was available for *vanA* or *blaOXA-48*. The PCR products were electrophoresed in 1% agarose gels in 1 × TBE buffer, stained with SimplySafe (EurX, Gdansk, Poland) and visualized under UV light. Amplicon identity of targeted genes was inferred from the expected product size on agarose gels; sequencing or restriction digestion was not performed.

For 16S rRNA amplicon sequencing, DNA extracts were submitted to Macrogen Europe (Amsterdam, The Netherlands) for library preparation targeting the V3–V4 hypervariable region using primers 341F/806R, followed by paired-end sequencing on an Illumina MiSeq platform (2 × 300 bp). Raw sequences were quality-filtered, trimmed, denoised and clustered into operational taxonomic units (OTUs) in QIIME2 (v2023.5). Taxonomic assignment was performed using the SILVA database using a naive Bayes classifier trained on the V3–V4 region. Alpha-diversity indices (observed OTU richness, Shannon entropy H, Simpson dominanceλ, Pielou evenness J, and Margalef’s richness Dm) were calculated from rarefied OTU tables, generated in our earlier study of technical snow microbial communities in this system [41]. All 16S rRNA sequence data used in this study originate from that earlier work. A full description of the pipeline, quality metrics and rarefaction curves is provided in [41]. The sequences are available under the BioProject accession number PRJNA1336629 (NCBI SRA). While [41] reported taxonomic community composition, the diversity indices presented here, and their integration into the multivariate and cluster analyses, are original to this study.

### 4.6. Statistical and Bioinformatic Analyses

Statistical analyses were performed in Statistica (v. 13.3, TIBCO Software, Inc., Palo Alto, CA, USA) and Python 3.12 (scipy v. 1.17). Data normality was assessed by the Shapiro–Wilk test and because bacterial counts, ARG richness, antibiotic concentrations and resistance percentages did not follow a normal distribution, and group sizes were small and uneven, differences among compartments were tested with the Kruskal–Wallis test (for bacterial counts, ARG richness and antibiotic concentrations) or with a permutation test based on 20,000 resamples (for resistance percentages, expressed as n/N). When a test showed an overall difference (*p* < 0.05), we compared compartments pairwise using Dunn’s test (for counts and concentrations) or Fisher’s exact test (for resistance percentages) and corrected the *p*-values for multiple comparisons with the Benjamini–Hochberg method. Each sample (one compartment, one chain, one sampling date) was treated as one data point. Compartments with fewer than three samples (for counts and concentrations) or fewer than five tested isolates (for resistance percentages) were not included in these tests and are instead described only with their median and range, or as raw counts. This applies to outflow water throughout, which was sampled only once. Full test results and *p*-values for every variable are provided in Supplementary Table S1. Pearson correlation was used to check relationships between continuous variables. A significance level of *p* < 0.05 was used throughout the tests.

Principal component analysis (PCA) was performed on a simplified dataset of nine system compartments (averaged across chains and seasons), given the sparse and unevenly distributed sample-level data available for several variables. Because this approach necessarily involves a lower number of observations than variables, the resulting ordination is presented as exploratory rather than confirmatory, and its interpretation is restricted to broad, descriptive gradients rather than formal statistical inference. The input matrix comprised 21 variables: ARG richness, ESBL prevalence, *Enterobacteriaceae* and *Aeromonas* resistance phenotype frequencies (beta-lactams, trimethoprim/sulfamethoxazole), normalized intensities of eleven antibiotic compounds (cefoxitin, erythromycin, tylosin, clindamycin, ofloxacin, ciprofloxacin, enrofloxacin, trimethoprim, sulfamethoxazole, linezolid, vancomycin), *E. coli* counts, *E. faecalis* counts, Shannon diversity index H and Margalef’s richness Dm. All variables were z-score standardized prior to PCA. Antibiotic intensities were normalized to the maximum observed value per compound across all sites and seasons before standardization, placing them on a dimensionless 0–1 scale comparable to proportional resistance data.

Hierarchical cluster analysis was performed on the same nine-compartment, z-scored dataset, using single linkage (nearest-neighbor method) applied to Euclidean distances (Statistica v. 13.3, TIBCO Software, Inc., Palo Alto, CA, USA).

## 5. Conclusions

This study provides the first comprehensive analysis of fate of AMR components, such as antibiotic residues, ARB and ARGs, throughout a complete snowmaking infrastructure, demonstrating that such systems can simultaneously act as barriers and/or vectors for the resistance determinants. The balance between these roles depends on individual indicators, infrastructure elements and the way of their handling.

Wastewater-affected source water introduces AMR micropollutants into the snowmaking systems. These micropollutants include antibiotics, ESBL-producing *Enterobacteriaceae* and ARGs that conventional biological treatment does not fully eliminate. Storage reservoirs can be considered infrastructure elements of a dual nature, where biological AMR indicators are reduced while antimicrobials are concentrated above the water intake or WWTP levels. Such an increase is attributed to solid particle adsorption, long hydraulic residence, protection from photodegradation, or oxidation in anoxic niches and refers to a variety of antimicrobials, including these of veterinary origin, entering the system through diffuse sources. Filtration infrastructure functioned as an additional retention barrier in this system, suggesting that engineering decisions and maintenance practices in snowmaking systems can affect the AMR fate therein. Technical snow and its ageing process further contributed to AMR reduction, with clearly lower antibiotic concentrations and lack of resistance gene detection, likely due to UV light exposure, freeze–thaw cell membrane disruption and ecological succession. Finally, reservoir sediments function as long-term ARG accumulation niches, preserving a variety of genes that can be further transferred horizontally. As such, they require careful consideration in terms of management and sediment disposal protocols.

Overall, this study demonstrates that snowmaking infrastructure can simultaneously act as an AMR barrier and/or its vector. The four zones proposed here (i.e., the contamination input zone, reservoir enrichment, transformation and reduction in snow cannon filters, technical snow, and AMR accumulation in sediments) provides a base that should be validated in broader range of ski resorts with various source water types, climate conditions and altitude. Nevertheless, incorporating snowmaking systems into environmental AMR monitoring, One Health risk assessments and winter tourism management strategies should be taken into consideration. This is important in terms of the direct recreational and occupational exposure of large numbers of people to technical snow at sites where wastewater-impacted water is the snowmaking source.

Finally, our findings, together with prior work in this catchment, point to insufficiently treated wastewater as a major contributor to AMR contamination at the snowmaking input sites. Addressing this upstream source, along with snowmaking chain monitoring, is a reasonable way of addressing the issue of AMR spread through this system.

## Figures and Tables

**Table 1:** Predominant antibiotic resistance types detected in the bacterial strains isolated along the snowmaking chain.

System Stage	No. of Isolates	Dominant Bacterial Groups	Predominant Resistance Phenotypes
WWTP effluent	24	*Aeromonas* (12) *Enterobacteriaceae* (12)	β-lactams (AMP 83.3%, 10/12) ESBL (33.3%, 4/12)
Intake water	31	*Aeromonas* (20) *Enterobacteriaceae* (11)	β-lactams (AMC 36.4%, 4/11) ESBL (72.7%, 8/11)
Reservoir water	42	*Aeromonas* (28) *Enterobacteriaceae* (6) *Acinetobacter* (4) *Pseudomonas* (3) Environmental cocci (1)	β-lactams (AMP 50% 3/6)
Snow cannon filters	32	*Enterobacteriaceae* (13) *Aeromonas* (8) *Acinetobacter* (4) Environmental cocci (2) and bacilli (5)	β-lactams (AMP 69.2%, 9/13) fluoroquinolones
Fresh technical snow	9	*Enterobacteriaceae* (3) *Acinetobacter* (3) *Aeromonas* (1) *Pseudomonas* (1) Environmental cocci (1)	β-lactams (AMP 66.7%, 2/3)
Aged snowpack	7	*Enterobacteriaceae* (3) *Pseudomonas* (1) Environmental cocci (1) and bacilli (2)	β-lactams (AMP 66.7%, 2/3)
Snowmelt	10	*Enterobacteriaceae* (7) *Aeromonas* (1) *Pseudomonas* (2)	β-lactams (AMC 28.6%, 2/7)
Reservoir sediment	21	*Enterobacteriaceae* (2) *Pseudomonas* (1) *Aeromonas* (11) Environmental bacilli (7)	none

## Data Availability

The original contributions presented in this study are included in the article/Supplementary Materials. Further inquiries can be directed to the corresponding author.

## Supplementary Materials

The following supporting information can be downloaded at https://www.mdpi.com/article/10.3390/ijms27188172/s1 [22,42,43,44,45,46,47,48,49,50,51,52,53,54,55,56,57,58,59,60,61,62].
