# Supplementary material for: Snowmaking Infrastructure: A Selective Barrier or Potential Vector for Antibiotic Residues and Resistance Determinants in Mountain Catchments
Source: Int J Mol Sci. 2026 Sep 14;27(18):8172. doi: 10.3390/ijms27188172 (PMC13607814; doi:10.3390/ijms27188172)
Supplement: Supplementary file 1 [file ijms-27-08172-s001.zip › Supplementary Table S2_Primers.pdf]

Supplementary Table S2. PCR primers for antimicrobial resistance genes with primer annealing temperature and product length

No.	Resistance Mechanism	Gene	Primer	Sequence (5′-3′)	Annealing Temp. (°C)	Product Length (bp)	Reference
1.	cephalosporinases	AmpC	AmpC-F	AATGGGTTTTCTACGGTCTG	50	191	[42]
			AmpC-R	GGGCAGCAAATGTGGAGCAA			
2.	beta-lactamases	blaPSE	blaPSE-F	GCGATCGGTTCGAATGAT	55	321	[43]
			blaPSE-R	ATTGAAGCCTGTGTTTGAGC			
3.	Extended-spectrum beta-lactamases (ESBL)	*blaTEM*	blaTEM-F	ATTCTTGAAGACGAAAGGGC	60	1150	[44]
			blaTEM-R	ACGCTCAGTGGAACGAAAAC			
4.		*blaCTX-M*	blaCTX-M-F	CGATGTGCAGTACCAGTAA	55	585	[45]
			blaCTX-M-R	TTAGTGACCAGAATCAGCGG			
5.		*bla-CTX-M1*	blaCTX-M1-F	AAAAATCACTGCGCCAGTTC	60	415	[46]
			blaCTX-M1-R	AGCTTATTCATCGCCACGTT			
6.		*blaCTX-M9*	blaCTX-M9-F	GTGACAAAGAGAGTGCAACGG	54	856	[47]
			blaCTX-M9-R	ATGATTCTCGCCGCTGAAGCC			
7.		*blaSHV*	blaSHV-F	CACTCAAGGATGTATTGTG	52	885	[44]
			blaSHV-R	TTAGCGTTGCCAGTGCTCG			
8.		*blaOXA-1*	blaOXA-1-F	ACACAATACATATCAACTTCGC	61	813	[44]
			blaOXA-1-R	AGTGTGTTTAGAATGGTGATC			
9.	Carbapenemases class D	*blaOXA-48*	blaOXA-48-F	GCTTGATCGCCCTCGATT	60	281	[48]
			blaOXA-48-R	GATTTGCTCCGTGGCCGAAA			
10.		*blaVIM*	blaVIM-F	GATGGTGTTTGGTCGCATA	60	390	[48]
			blaVIM-R	CGAATGCGCAGCACCAG			
11.		*blaNDM*	blaNDM-F	GGTTTGGCGATCTGGTTTTC	60	621	[49]
			blaNDM-R	CGGAATGGCTCATCACGATC			
12.	Methicillin-resistance	*mecA*	mecA-F	GTAGAAAATGACTGAACGTCCGATAA	55	310	[50]
			mecA-R	CCAATTCCACATTGTTTCGGTCTAA			
13.	Macrolide–lincosamide–streptogramin B (MLSb) resistance genes	*ereB*	ereB-F<br>ereB-R	AGAAATGGAGGTTCATACTTACCA<br>CATATAATCATCACCAATGGCA	52	546	[51]
14.		*ermA*	ermA-F<br>ermA-R	TCTAAAAAGCATGTAAAAGAA<br>CTTCGATAGTTTATTAATATTAGT	52	645	[51]
15.		*ermB*	ermB-F	GAAAAGGTACTCAACCAAATA	55	639	[51]

			ermB-R	AGTAACGGTACTTAAATTGTTTAC			
16.		*ermC*	ermC-F<br>ermC-R	ATCTTTGAAATCGGCTCAGG<br>CAAACCCGTATTCCACGATT	52	295	[52]
17.		*msrA*	msrA-F<br>msrA-R	GGCACAATAAGAGTGTTTAAAGG<br>AAGTTATATCATGAATAGATTGTCCTGTT	50	940	[53]
18.		*msrB*	msrB-F<br>msrB-R	TATGATATCCATAATAATTATCCAATC<br>AAGTTATATCATGAATAGATTGTCCTGTT	50	595	[53]
19.		*mphA*	mphA-F<br>mphA-R	AACTGTACGCACTTGC<br>GGTACTCTTCGTTACC	50	837	[51]
20.		*mphC*	mphC-F<br>mphC-R	GAGACTACCAAGAAGACCTGACG<br>CATACGCCGATTCTCCTGAT	60	722	[54]
21.		*vatA*	vatA-F<br>vatA-R	CAATGACCATGGACCTGATC<br>CTTCAGCATTTCGATATCTC	52	619	[53]
22.		*vatB*	vatB-F<br>vatB -R	CCCTGATCCAAATAGCATATATCC<br>CTAAATCAGAGCTACAAAGT	52	602	[53]
23.		*vga*	vga-F<br>vga-R	CCAGAACTGCTATTAGCAGATGAA<br>AAGTTCGTTTCTCTTTTCGACG	54	470	[53]
24.		*vgb*	vgb-F<br>vgb-R	ACTAACCAAGATACAGGACC<br>TTATTGCTTGTCAGCCTTCC	53	734	[53]
25.	*Streptomycin resistance*	*strA*	strA-F<br>strA-R	TCAATCCCGACTTCTTACCG<br>CACCATGGCAAACAACCATA	52	126	[55]
26.	*Trimetophrim resistance*	*dfrA12*	dfrA12-F<br>dfrA12-R	TTTATCTCGTTGCTGCGATG<br>AGGCTTGCCGATAGACTCAA	50	155	[22]
27.	*Aminoglycoside resistance*	aac(6')/aph(2')	aac(6')/aph(2'')-F<br>aac(6')/aph(2'')-R	CAGAGCCTTGGGAAGATGAAG<br>CCTCGTGTAATTCATGTTCTGGC	55	348	[56]
28.	*Tetracyclines resistance*	*tetK*	tetK-F<br>tetK-R	TCGATAGGAACAGCAGTA<br>CAGCAGATCCTACTCCTT	55	169	[57]
29.		*tetL*	tetL-F<br>tetL-R	TCGTTAGCGTGCTGTCATTC<br>TGTAACTCCGATGTTTAACACG	58	628	[58]
30.	*Sulfonamides resistance*	*sul1*	sul1-F<br>sul1-R	CTTCGATGAGAGCCGGCGGC<br>GCAAGGCGGAAACCCGCGCC	56	650	[59]
31.		*sul2*	sul2-F<br>sul2-R	CGCTCAAGGCAGATGGCATT<br>GCGTTTGATACCGGCACCCGT	56	250	[59]

32.		*sul3*	sul3-F<br>sul3-R	ACCACCGATAGTTTTTCCGA<br>TGCCTTTTTCTTTTAAAGCC	62	199	[22]
33.	*Fluoroquinolone resistance*	*qnrA*	qnrA-F<br>qnrA-R	ATTTCTCACGCCAGGATTTG<br>GATCGGCAAAGGTTAGGTCA	53	516	[60]
34.		*qnrB*	qnrB-F<br>qnrB-R	GATCGTGAAAGCCAGAAAGG<br>ACGATGCCTGGTAGTTGTCC	53	469	[60]
35.		*qnrD*	qnrD-F<br>qnrD-R	CGAGATCAATTTACGGGGAATA<br>AACAAGCTGAAGCGCCTG	53	582	[61]
36.		*qnrS*	qnrS-F<br>qnrS-R	ACGACATTCGTCAACTGCAA<br>TAAATTGGCACCCTGTAGGC	53	417	[60]
37.	*Vancomycin resistance*	*vanA*	vanA-F<br>vanA-R	GGGAAAACGACAATTGC<br>GTACAATGCGGCCGTTA	52	732	[62]
